# Quantification of the coupled dynamics of marine microbes and reactive oxygen species in laboratory batch culture experiments

**DOI:** 10.1128/spectrum.00712-25

**Published:** 2026-05-12

**Authors:** Donna K. McCullough, Emily C. Bowden, Benjamin C. Calfee, Michael A. Gilchrist, Erik R. Zinser, David Talmy

**Affiliations:** 1 Department of Microbiology, University of Tennessee 189504, Knoxville, Tennessee, USA; 2 Department of Ecology and Evolutionary Biology, University of Tennessee 189505, Knoxville, Tennessee, USA; University of Mississippi, University, Mississippi, USA

**Keywords:** reactive oxygen species, hydrogen peroxide, cyanobacteria, mathematical modeling

## Abstract

*Prochlorococcus*, the smallest and most numerous phytoplankter of the oligotrophic surface ocean, is vulnerable to damage by the reactive oxygen species hydrogen peroxide (H_2_O_2_). The most harmful effects of exogenous H_2_O_2_ are mitigated by detoxifying organisms, but even at low concentrations, the ecological and biogeochemical consequences of H_2_O_2_ damage are not fully understood. Here, we present coupled ordinary differential equation models of the dynamics of diverse marine microbes grown in different levels of H_2_O_2_. We fit our model to monoculture time-series data of peroxide-sensitive *Prochlorococcus* and peroxide-resistant *Synechococcus*, picoeukaryotes, and bacteria. Our fits provide estimates of H_2_O_2_ cell damage and detoxification rates in these ecologically important microbes. We find significant variation in rates of cell death and H_2_O_2_ detoxification by taxonomy and H_2_O_2_ concentration. Picoplankton (*Micromonas* spp. or *Ostreococcus* spp.) and other previously investigated heterotroph helpers (*Alteromonas*) appear to upregulate detoxification when H_2_O_2_ is elevated, whereas *Synechococcus* detoxification rate does not vary with hydrogen peroxide concentration. As expected, elevated H_2_O_2_ is damaging to high light-adapted *Prochlorococcus*, but they are capable of modest detoxification when H_2_O_2_ concentration is relatively low. Our use of differential equation models to quantify parameters underlying microbe-H_2_O_2_ dynamics provides a basis for ecosystem models to explore the impact of reactive oxygen species on cell mortality of different ocean microbes and computational exploration of H_2_O_2_ impacts on microbial community composition.

**IMPORTANCE:**

Hydrogen peroxide produced naturally in the surface ocean is toxic to many abundant microbes that perform important ecosystem services such as carbon fixation. Much of the naturally occurring hydrogen peroxide produced in the surface ocean is detoxified by “helper” organisms. The healthy function of microbial communities may therefore depend upon the presence of different helpers. Mathematical models provide an important means to evaluate the impacts of hydrogen peroxide on ecosystem function. This study introduces a framework to evaluate the sensitivity of diverse microbes to hydrogen peroxide, in a manner that is amenable for inclusion in ecosystem models. It lays a foundation for future efforts to evaluate the impact of hydrogen peroxide on ecosystem function and microbial community composition.

## INTRODUCTION

Two globally dominant genera of cyanobacteria, *Prochlorococcus* and *Synechococcus* perform up to ~50% of marine carbon fixation (1–4). *Prochlorococcus* is the most numerous phytoplankter (5–9) and numerically dwarfs its sister genus, *Synechococcus*, in nutrient-deplete gyres (10). Predictions of future *Prochlorococcus* distributions, despite altered climate considerations and shifting environmental pressures (11, 12), indicate that *Prochlorococcus* spp. may remain numerically dominant at mid to low latitudes throughout much of the surface open ocean (4). The numerical dominance of *Prochlorococcus* in these areas is thought to be due to its minimal nutrient requirements and high surface area to volume ratio associated with its smaller cell size (0.6–0.8 μm vs 0.6–2.0 μm) and total genome lengths (1.5–2.5 million base pairs [Mbp] vs >2.4 Mbp) compared to *Synechococcus* (13–15).

One striking aspect of *Prochlorococcus*’s streamlined genome is its evolutionary loss of catalase, a key enzyme in the protection against the reactive oxygen species (ROS) hydrogen peroxide (H_2_O_2_) that is present in surface ocean environments (16–20). Reactive oxygen species are an inevitable side effect of marine aerobic life (21–24). ROS can arise from biotic processes like photosynthesis (25–27) and respiration (28, 29), as well as from abiotic processes such as photocatalytic H_2_O_2_ production via sunlight and dissolved organic carbon (DOC) (30–32). H_2_O_2_ is created abiotically when extracellular chromatic dissolved organic material absorbs ultraviolet and visible light, leading to transfer of electrons to dissolved oxygen in the surface ocean (30, 33, 34), and from precipitation events occurring over aquatic environments (35, 36). H_2_O_2_, unlike most other reactive oxygen species, is uncharged and thus can readily diffuse across cell membranes (22, 29, 37–39). *Prochlorococcus*’s lack of catalase (32, 34, 40) is even more striking, given that abiotic production from a single diel cycle produces enough H_2_O_2_ to kill *Prochlorococcus* (19). Genera with catalases therefore provide a benefit to their community through exogenous H_2_O_2_ detoxification (19, 22, 41, 42).

**Fig 1:**
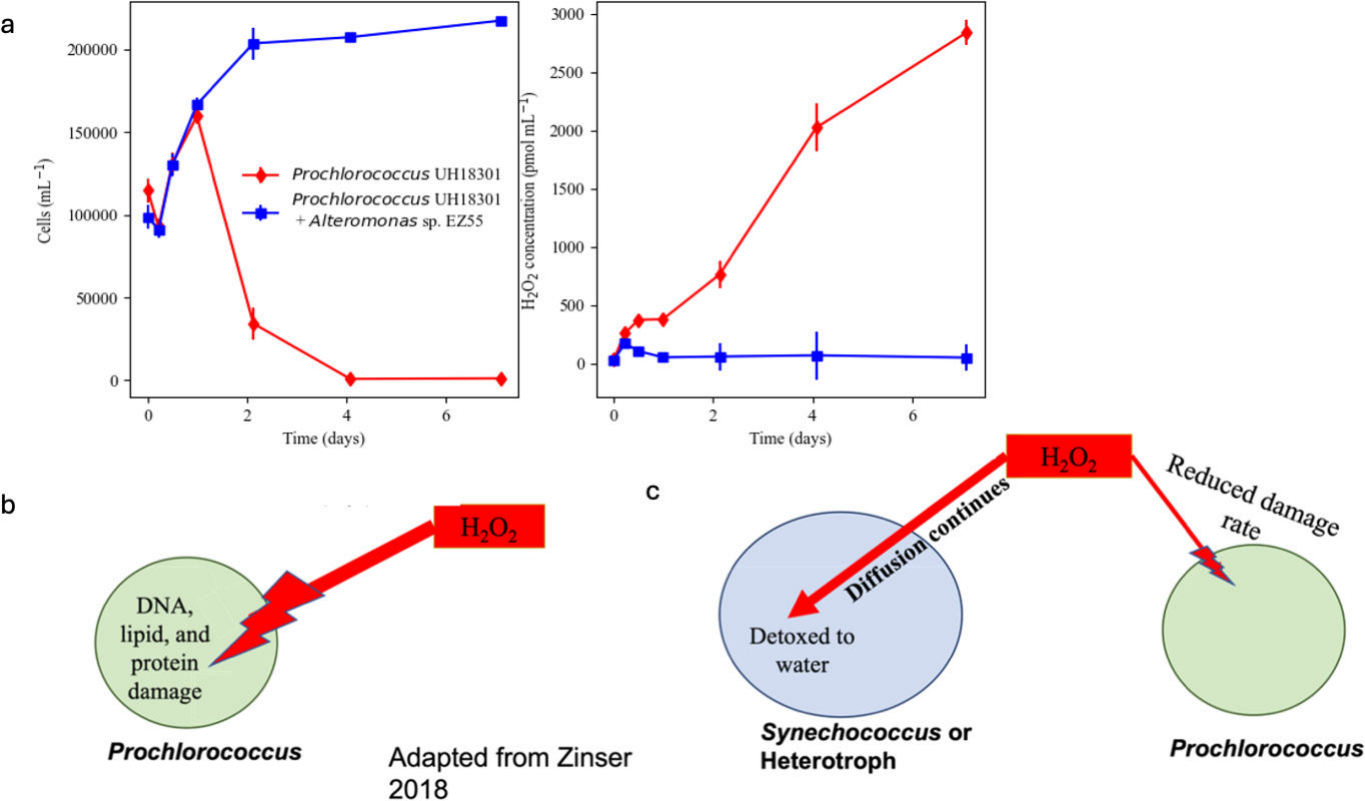
Prochlorococcus growth is dependent on the presence of detoxifying organisms. (**a**) Prochlorococcus abundance (left) and H_2_O_2_ concentrations (right) in batch cultures with photochemical H_2_O_2_ production with (blue) and without (red) *Alteromonas* EZ55, a heterotrophic bacterial helper. Data are from reference 19. (**b**) Schematic depiction of H_2_O_2_ diffusion across cell membranes leading to DNA, lipid, and protein damage in organisms lacking catalase, such as *Prochlorococcus*. (**c**) Schematic representation of detoxifying “helpers.”

With the inherent leakiness of H_2_O_2_ across membranes (43, 44), if an organism detoxifies intracellular H_2_O_2_, the laws of diffusion and chemical reaction balance will dictate that the internal absence of H_2_O_2_ will draw exogenous H_2_O_2_ into the cell (38). Therefore, detoxification of H_2_O_2_ cannot be just for one cell and potentially benefits the entire local microbial community. This is typically described as a “leaky function” (30, 45, 46). Without the ability to detoxify H_2_O_2_ efficiently, *Prochlorococcus* is dependent on other species for this vital detoxification function (19, 22, 47, 48). Various studies have shown *Prochlorococcus* spp. that did not retain catalase are unable to survive environmentally relevant H_2_O_2_ production without detoxifying species (like *Synechococcus*) or heterotrophic bacteria (like *Alteromonas* spp.) (17, 18, 22, 42, 49).

Ecosystem (50, 51) and statistical niche models (4, 52) calculate plankton abundances differently, but both types of models indicate that *Prochlorococcus* dominates in the most oligotrophic regions of the ocean (4, 53). Realistic patterns of *Prochlorococcus* biogeography are recreated in ecosystem models that assume *Prochlorococcus* has superior competitive ability for ammonium (53, 54). More recent modeling studies indicate that *Prochlorococcus* loss processes, such as viral predation and grazing, are also important influences on *Prochlorococcus* biogeography (55, 56). Despite recognition that *Prochlorococcus* biogeographic range is critically dependent on the presence of detoxifying helpers (42), the role of H_2_O_2_ dynamics on dominant oceanic microbial abundances has not been assessed with ocean models.

Here, we present a coupled ordinary differential equation (ODE) model for microbial and H_2_O_2_ dynamics. As our model illustrates, coupled ODEs are well suited to quantification of cell-H_2_O_2_ dynamics. As microbial populations grow and die, their ability to detoxify exogenous reactive oxygen species changes. Our ODE model explicitly quantifies changes in population sizes and H_2_O_2_ concentration. By fitting our model to laboratory time-series data using a Bayesian Monte Carlo Markov chain (MCMC) approach, we obtain estimates of cell-specific death and detoxification rates for globally important genera. Finally, we discuss how our ODE model can be used to explore the impact of H_2_O_2_ on cell mortality and ecosystem function.

## MATERIALS AND METHODS

Below we describe the system of ODEs we used to model the population dynamics of bacteria across a range of H_2_O_2_ concentrations. Special cases of our model were fitted in different permutations of laboratory community assays using a Bayesian Metropolis-Hastings MCMC to estimate model parameter posterior distributions (see Table 1 for definitions of all parameters) (57). All computer code is provided in a GitHub repository (https://github.com/DKMcCullough/HOOH_dynamics). Below is a detailed description of both the model equations and data used to estimate model parameters.

**TABLE 1:** Parameter and variable definitions and units

Symbol	Description	Units
$\mu_{i}$	Growth rate	day^−1^
$\kappa_{dam,i}$	H_2_O_2_-specific rate of cell death	mL pmol^−1^ day^1^
$S_{H}$	H_2_O_2_ supply rate	pmol mL^−1^ day^−1^
$\delta_{H}$	H_2_O_2_ decay rate	day^−1^
$\phi_{det,i}$	Cell-specific rate of H_2_O_2_ detoxification	mL cell^−1^ day^−1^
$P_{i}$	Cell abundance	cells mL^−1^
H	H_2_O_2_ concentration	pmol mL^−1^

### Model equations

The dynamics of phytoplankton, *i*, abundance ($P_{i}$, cells mL^−1^), and H_2_O_2_ concentration (*H*, pmol mL^−1^) were modeled with a set of coupled ODEs:


(1) $$\begin{array}{rl} \frac{dP_{i}}{dt} & =\underset{\text{net growth}}{\underbrace{\mu_{i}P_{i}}}-\;\underset{\text{cell death}}{\underbrace{\kappa_{dam,i}P_{i}H}}\\ \frac{dH}{dt} & =\underset{\begin{array}{c} \text{hydrogen}\\ \text{peroxide}\\ \text{supply rate} \end{array}}{\underbrace{S_{H}}}-\underset{\text{abiotic decay}}{\underbrace{\delta_{H}H}}-\underset{\text{biotic detoxification}}{\underbrace{\phi_{det,i}P_{i}H}} \end{array}.$$

All parameter and state variable definitions and units are provided in Table 1. Phytoplankton grow at a constant rate $\mu_{i}$ (day^−1^). The main rate of cell death is assumed to be dependent on H_2_O_2_ concentration and a mass action coefficient, $\kappa_{dam,i}$ (mL pmol^−1^ day^−1^), connecting cell death to H_2_O_2_ concentration. H_2_O_2_ is supplied at rate *S_H_* (pmol mL^−1^ day^−1^) and subject to first-order abiotic decay at the rate $\delta_{H}$ (day^−1^), and cell contact-dependent detoxification is set by the mass action coefficient $\phi_{det,i}$ (mL cell^−1^ day^−1^), connecting cell concentration and H_2_O_2_ loss.

Note that cellular production of H_2_O_2_ is assumed to be negligible, as the experiments were all conducted under low light conditions. Furthermore, we interpret cell-mediated H_2_O_2_ loss to be due to enzymatic detoxification but note that oxidation by a range of macromolecules is possible (58), and the model does not distinguish between these alternate pathways.

### Laboratory data

Original data, along with methods for hydrogen peroxide measurement and culturing, are described in (59). Briefly, exogenous H_2_O_2_ concentration was measured using an acridinium ester chemiluminescence injection protocol (19, 60) in abiotic controls and growth media. Each luminescence reading was transformed to H_2_O_2_ pmol mL^−1^ data via a reference curve made in Microsoft Excel following Morris and Zinser (61) and Morris et al. (19) protocols. This was done for readings from both a spike-free control (0 pmol mL^−1^ H_2_O_2_ added) and spiked (400 pmol mL^−1^ H_2_O_2_ added) assays for abiotic and plankton media samples. All cyanobacterial stock cultures were maintained in an artificial seawater medium; however, picoeukaryote stock cultures were maintained, and all experiments were performed using an AMP-A derivative, AMP-PE, which allowed for efficient and consistent growth of all photosynthetic microbes in mono- and coculture. Cell densities of *Prochlorococcus* and *Synechococcus* were measured using different autofluorescent properties in flow cytometric assays (62, 63). Growth curves in 0 or 400 pmol mL^−1^ H_2_O_2_ spikes for picoeukaryote genera *Micromonas* (and 300 and 800 pmol mL^−1^ for *Alteromonas macleodii*, EZ55) were then considered. Following published methods, heterotroph cells were counted through plating assays as concurrent H_2_O_2_ measurements were taken (63). Note that only exponential phase data were used for model fitting. All biotic data can be found at the Biological and Chemical Oceanography Data Management Office (64).

### Fitting procedure

Our ODE model as defined in Equation 1 has five unknown parameters: plankton growth rate ($\mu_{i}$), H_2_O_2_-induced cellular death rate ($\kappa_{dam,i}$), cell-mediated H_2_O_2_ detoxification rate ($\phi_{det,i}$), abiotic H_2_O_2_ supply rate (*S_H_*), and abiotic H_2_O_2_ decay rate ($\delta_{H}$). The cell and H_2_O_2_ concentration (*H_0_* and *P_i,0_*, respectively) at the start of each experiment were also considered fitting parameters. Our goal was to find the combination of parameters that best explained observed H_2_O_2_ and cell abundance dynamics in each laboratory population data set. We chose to use the Metropolis-Hastings algorithm (57, 65), a widely used MCMC method. We took a Bayesian approach to parameter quantification, thereby assuming that each parameter is itself a distribution with inherent uncertainty. A summary of our key assumptions of the fitting procedure is included below. A full explanation of the fitting procedure is included in Supplemental material.

The goal of our model fitting was to sample and summarize the posterior probability distribution of the model parameters, given the model and data. Let *D* be a given experimental data set and let $\theta_{i}$ be the set of parameters of interest (e.g., for the full model $\theta_{i}$ = $\left\{\mu_{i},\kappa_{dam,i},\ldots \right\}$). The following describes a procedure to estimate the posterior probability distributions of the parameter $\theta_{i}$, given the data *D*, or ${P(\theta }_{i}\text{|}D)$.

Using Bayes’ theorem, the probability of a given parameter set $\theta_{i}$, given data set *D*, is


(2) $$P\left(\theta_{i}\text{|}D\right)=\frac{P\left(D\text{|}\theta_{i}\right)P\left(\theta_{i}\right)}{P\left(D\right)},$$

where *P*($\theta_{i}$*)* represents the prior distribution for our parameters. For simplicity, we assumed uniform priors on all parameters, placing a lower bound at 10^−8^ to prevent the model from exploring parameters that are numerically indistinguishable from zero. Observational uncertainty and other noise in our data are generally distributed log normally around the “true” state of the system. Under this assumption of log(N) error, the likelihood function, $L\left(\theta_{i}\right)$, is defined as follows:


(3) $$P\left(D|\theta_{i}\right)=L\left(\theta_{i}\right)=exp\left(-\chi^{2}\right).$$

Using $y_{i,D}$ to represent data point *i*, such as H_2_O_2_ concentration or cell abundance, and $y_{i,\theta }$ to represent our model’s predicted value, given a set of parameter values $\theta_{i}$,


(4) $$P\left(D|\theta_{i}\right)\propto exp\left(\sum_{i}\frac{{\left(ln\left(y_{i,D}\right)-ln\left(y_{i,\theta }\right)\right)}^{2}}{\sigma_{y,i}^{2}}\right),$$

where $\sigma_{y,i}$ quantifies the noise in our measurements. For simplicity of our model fittings, we set $\sigma_{y,i}$ by analyzing the standard deviation of our log-transformed measurements across technical and biological replicates (see Supplemental material).

### Model implementation

Abiotic H_2_O_2_ production and decay parameters, *S_H_* and $\delta_{H}$, were constrained first via cell-free dynamics of hydrogen peroxide. These parameters were used in subsequent experiments to constrain the background, abiotic production of H_2_O_2_. Analogously, ordinary least squares regression was used to constrain growth rates, $\mu_{i}$ with spike-free log-transformed growth curves (Table 2). These growth rates were then assumed in all subsequent model fittings with a range of catalase-positive and catalase-negative organisms in both unspiked and H_2_O_2_-spiked media, leading to quantification of damage ($\kappa_{dam,i}$) and detoxification ($\phi_{det,i}$) rates (Fig. 1).

**TABLE 2:** Exponential growth rates fitted with ordinary least squares regression in spike-free conditions

Organism	Growth rate (day^−1^)
*Prochlorococcus* MIT 9215	0.25
*Synechococcus* WH7803	0.3
*Synechococcus* CC9605	0.36
*Micromonas comoda*	0.54
*Micromonas pusilla*	0.49
*Ostreococcus lucimarinus*	0.55
*Ostreococcus tauri*	0.74
*Alteromonas macleodii*	8.0

## RESULTS

**Fig 2:**
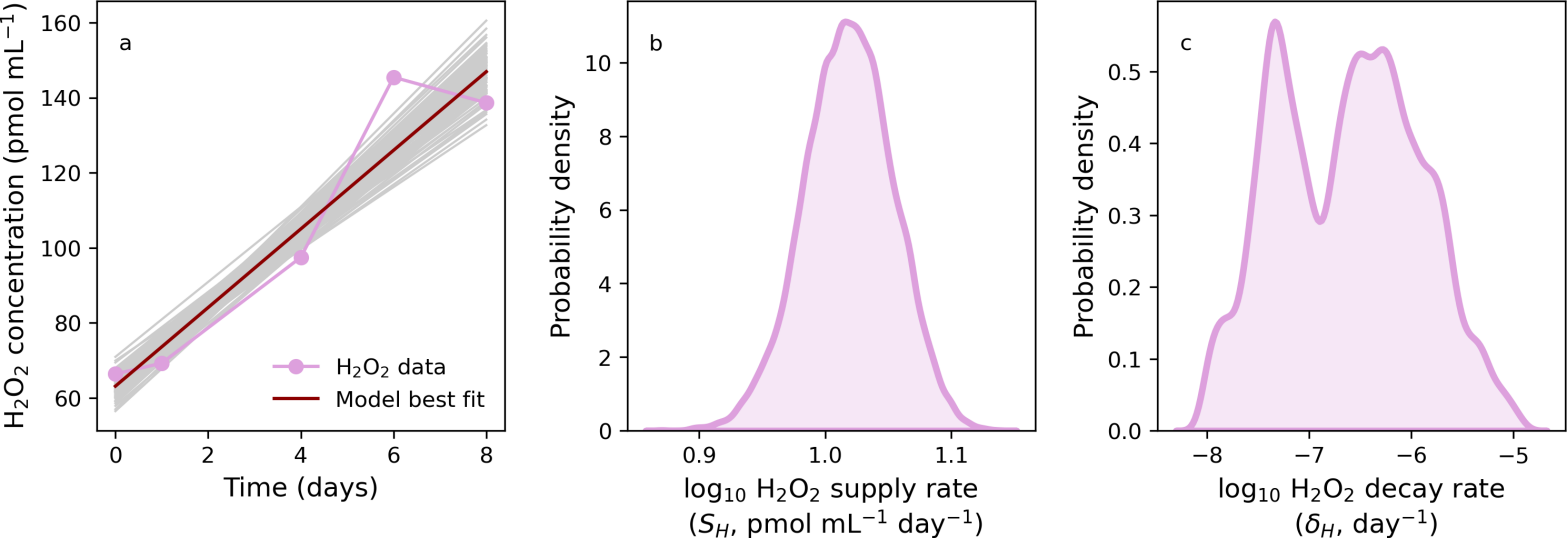
Modeling hydrogen peroxide dynamics in abiotic media with 0 pmol mL^−1^ H_2_O_2_ spike. (**a**) Laboratory H_2_O_2_ measurements (connected pink symbols), model fit (red line), and model uncertainty (gray lines). (**b and c**) Kernel density estimates representing uncertainty in H_2_O_2_ supply (**b**) and decay (**c**) rates. Note that here and elsewhere, probability density refers to the posterior density after log_10_ transformation.

In Fig. 2a, we present both the laboratory data (pink symbols) and model fit (red line) for H_2_O_2_ in cell-free medium with a zero additional input of H_2_O_2_. Here, we set *Prochlorococcus* cell concentrations (*P_i_*) to zero in Equation 1. In Fig. 2b and c, we present kernel density estimates that indicate the probability of various H_2_O_2_ supply and decay rates, given the time-course data in Fig. 2a. Our data and model both showed rising H_2_O_2_ concentration despite no H_2_O_2_ addition. This is consistent with prior studies indicating illuminated growth media produces H_2_O_2_ (61).

**Fig 3:**
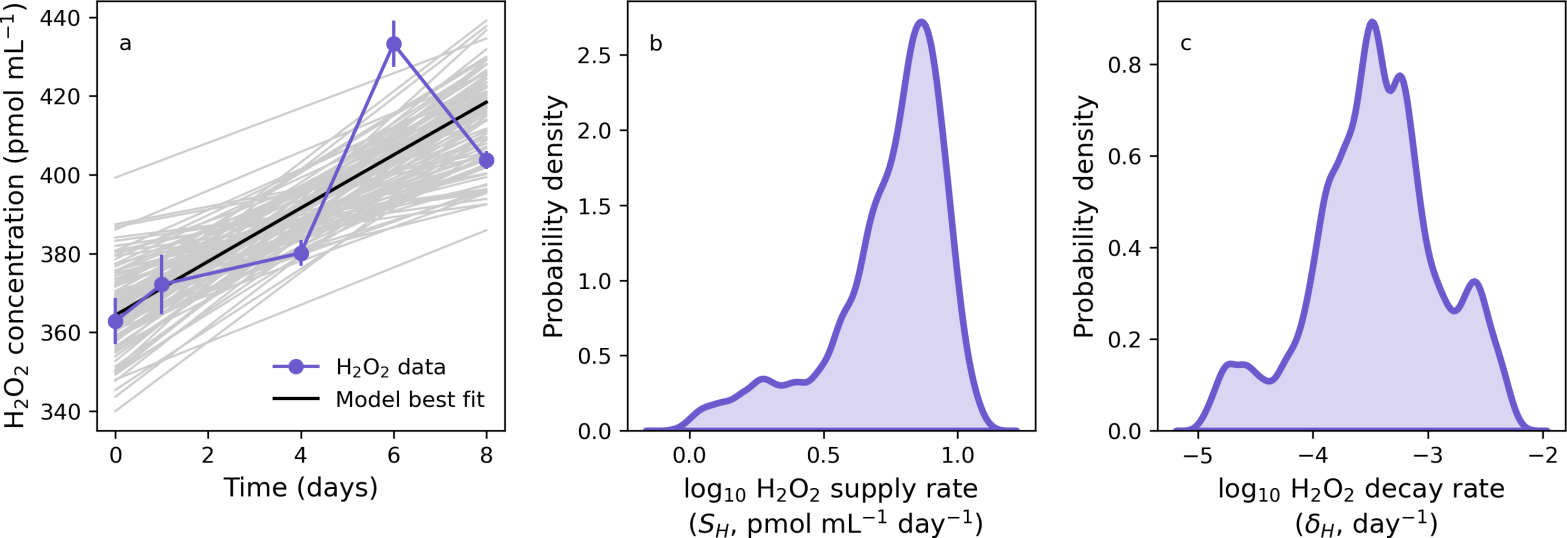
Modeling hydrogen peroxide dynamics in abiotic media with 400 pmol mL^−1^ H_2_O_2_ spike. (**a**) Laboratory H_2_O_2_ measurements (connected blue symbols), model fit (black line), and model uncertainty (gray lines). (b and c) Kernel density estimates representing uncertainty in estimates of H_2_O_2_ supply (**b**) and decay (**c**) rates.

Using the same cell-free model, we captured and represented H_2_O_2_ dynamics and parameter distributions for the 400 pmol mL^−1^ H_2_O_2_ spiked medium (Fig. 3). Our posterior samples for the abiotic production rate were slightly lower but within a similar range to the zero-addition treatment in Fig. 2. Across treatments, the decay rate was generally less than 1 × 10^−4^ day^−1^, which is negligible by comparison to rates of detoxification recorded in the environment, which typically exceed 1 × 10^−3^ hour^−1^ (66). Thus, our model captures H_2_O_2_ spike assay dynamics in cell-free culture conditions at multiple starting spike levels, providing an important constraint on the rate of H_2_O_2_ supply and decay in growth media. We use these parameter ranges to define background abiotic H_2_O_2_ dynamics when Equation 1 is later considered in the presence of microbial cells.

### Phytoplankton batch culture dynamics without amended hydrogen peroxide

**Fig 4:**
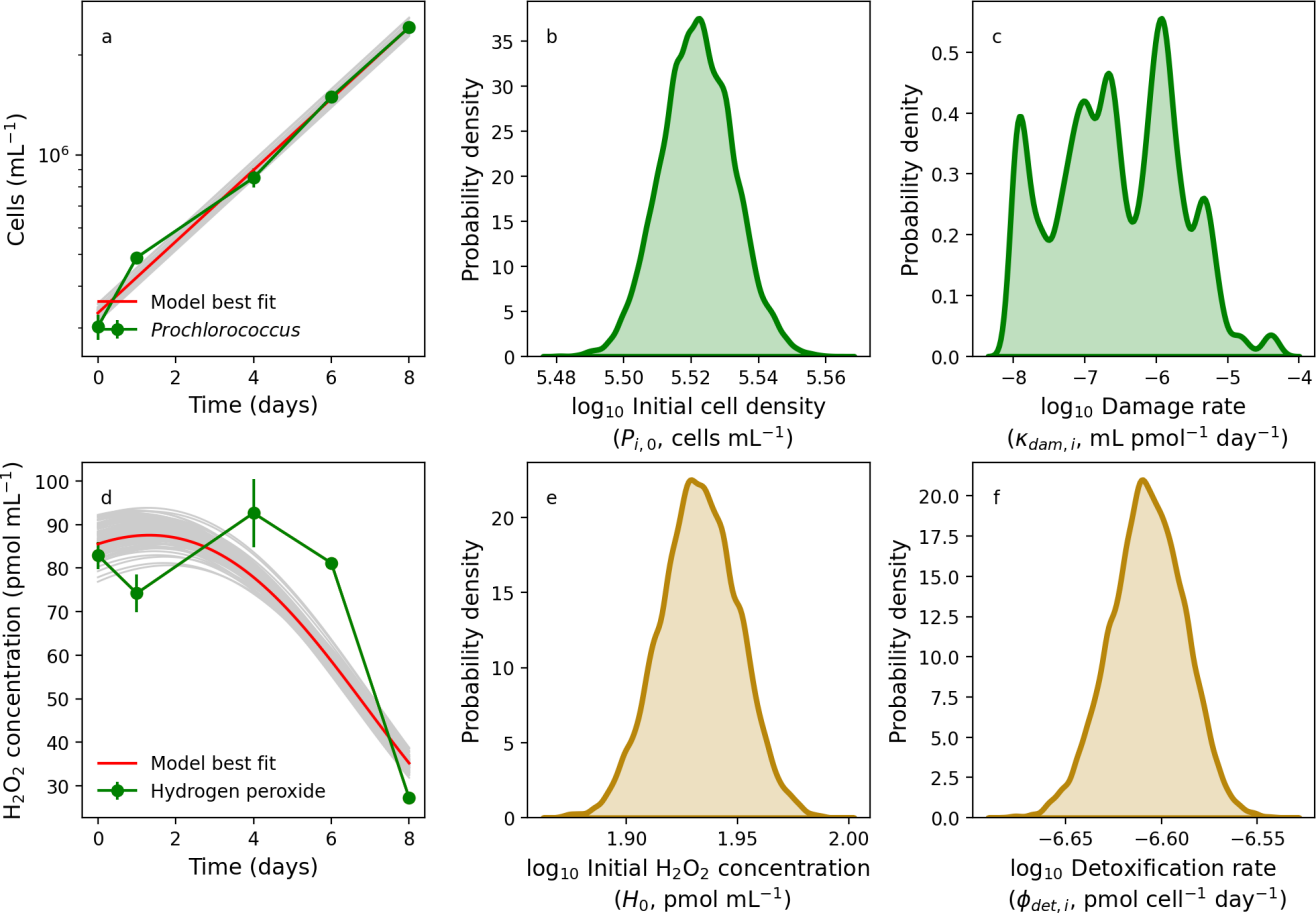
Modeling the growth of *Prochlorococcus* in monoculture. Laboratory batch culture population dynamics of *Prochlorococcus* (green line) and model searches (gray lines) with best-fit solution (red line) with 0 pmol mL^−1^ H_2_O_2_ addition (**a**), along with parameter posterior distributions for the initial population size (**b**) and the H_2_O_2_-mediated rate of cell death (**c**). Also shown is a model-data comparison of hydrogen peroxide dynamics (**d**), along with probability distributions for the initial H_2_O_2_ concentration (**e**) and the cell-specific rate of hydrogen peroxide detoxification (**f**).

Being now able to account for relevant abiotic processes, we next considered how microbial dynamics may be impacted by H_2_O_2_. Figure 4 shows our model trained and compared with *Prochlorococcus* strain MIT9215 in exponential growth. The model provides a satisfactory fit to the population density change over time, which clearly followed an exponential increase (Fig. 4a), leading to quantification of uncertainty in the initial cell density and damage rate (Fig. 4b and c). The H_2_O_2_-mediated damage rate varied over many orders of magnitude over a range that in practice is indistinguishable from zero (Fig. 4c). This is to be expected, because the growth rate assumed here was fit directly to the growth curve in Fig. 4a. Moreover, the model captured *Prochlorococcus*-mediated decline in H_2_O_2_ (Fig. 4d), leading to constraint on the initial H_2_O_2_ concentration (Fig. 4e) and *Prochlorococcus* detoxification rate (Fig. 4f).

### Phytoplankton batch culture dynamics with amended hydrogen peroxide

**Fig 5:**
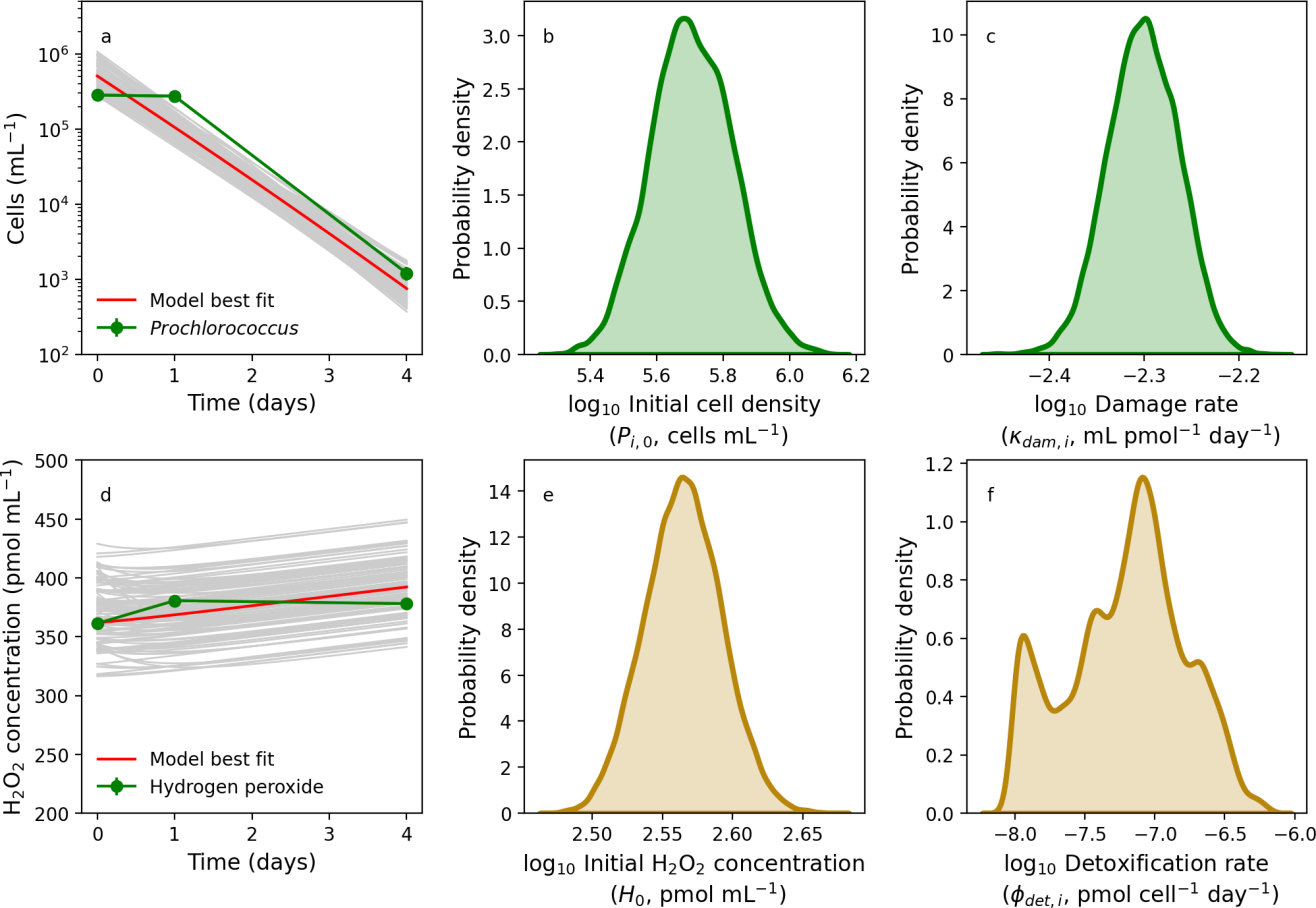
Monoculture *Prochlorococcus* modeled in H_2_O_2_ spiked media. Coupled laboratory batch culture dynamics of *Prochlorococcus* (**a**) and H_2_O_2_ (**d**) data alongside model best-fit (red line) and searched (gray) combinations in laboratory assays containing 400 pmol mL^−1^ H_2_O_2_. To the right of each dynamics plot, the initial value of *Prochlorococcus*, *P_i_*_*,0*_ (**b**), and hydrogen peroxide, *H*_*0*_ (**e**), and the controlling parameter for each line: damage rate *κ*_*dam*,_*_i_* (**c**) and detoxification rate *ϕ*_*det*,_*_i_* (**f**) are shown.

Having quantified *Prochlorococcus* growth without a fixed H_2_O_2_ addition, we then compared our mode in Equation 1 with experimental values for microbial abundance and H_2_O_2_ concentration. In Fig. 5, we present the dynamics of *Prochlorococcus* in media with a 400 pmol mL^−1^ spike of H_2_O_2_. Our results clearly indicate that *Prochlorococcus* died once H_2_O_2_ was added to the growth medium (Fig. 5a). This tracks with previous laboratory studies that signified *Prochlorococcus* cannot grow alone in exogenous H_2_O_2_ above 400 pmol mL^−1^ (19, 67, 68). We kept the previously quantified exponential growth rate $\mu_{i}$ from the model’s initial fitting of *Prochlorococcus* in zero H_2_O_2_. Having trained our model on cellular media with the addition of a H_2_O_2_ spike, we can quantify uncertainty in two H_2_O_2_-based parameters: the H_2_O_2_-specific rate of cell death, $\kappa_{dam,i}$ (Fig. 5c), and the cell-specific rate of detoxification, $\phi_{det,i}$ (Fig. 5f). Importantly, our model predicts vanishingly small values for *Prochlorococcus* $\phi_{det,i}$ in Fig. 5f, indicating negligible cell-based detoxification of exogenous H_2_O_2_.

### Modeling *Synechococcus* and H_2_O_2_ dynamics

**Fig 6:**
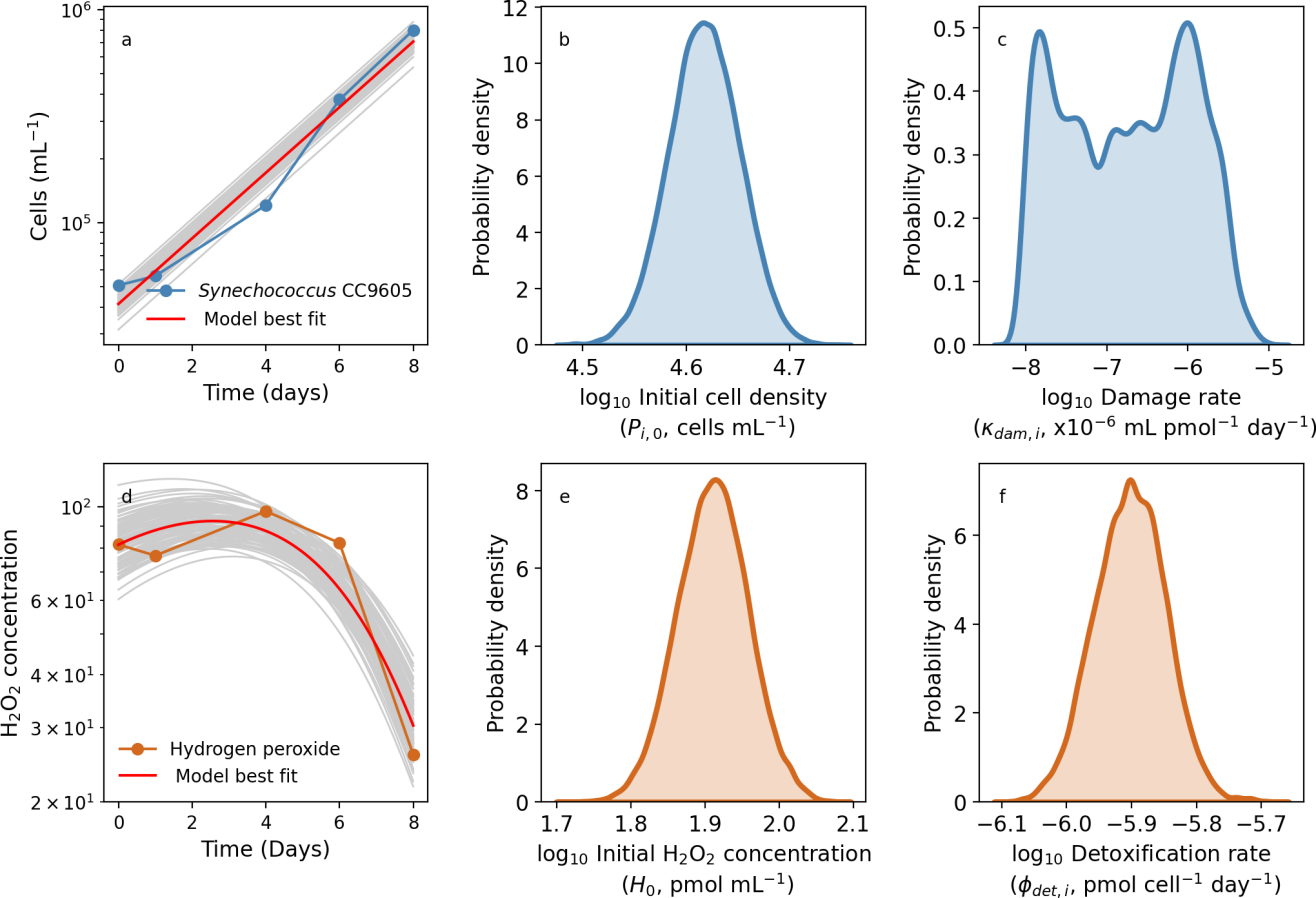
Monoculture *Synechococcus* CC9605 modeled in H_2_O_2_ unspiked media. Coupled laboratory batch culture dynamics of *Synechococcus* CC9605 (**a**) and H_2_O_2_ (**d**) data alongside model best-fit (red line) and searched (gray) combinations in laboratory assays containing no added H_2_O_2_. To the right of each dynamics plot, the initial value of *Synechococcus* CC9605, P_i,0_ (**b**) and hydrogen peroxide, *H*_0_ (**e**) and the controlling parameter for each line: damage rate *κ*_*dam*,*i*_ (**c**) and detoxification rate *ϕ*_*det*,*i*_ (**f**) are shown.

**Fig 7:**
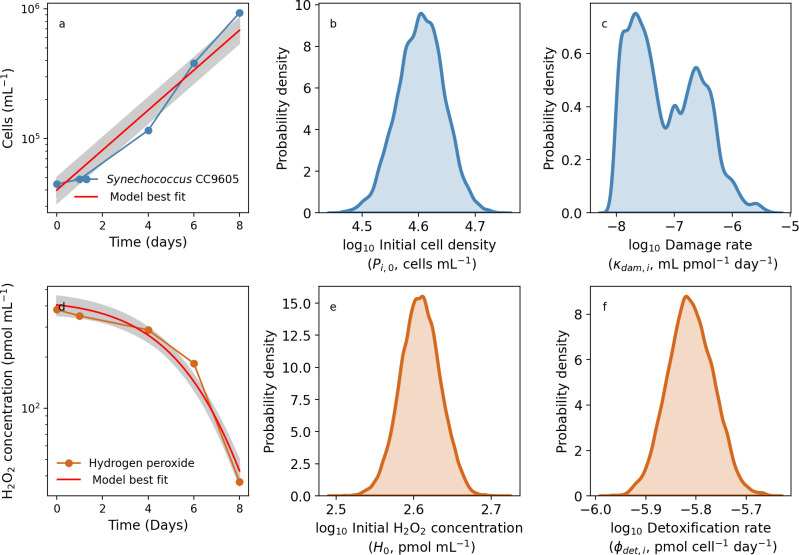
Coupled laboratory batch culture dynamics of *Synechococcus* CC9605 (**a**) and H_2_O_2_ (**d**) data alongside searched parameter combinations (gray) in batch culture containing 400 pmol mL^−1^ H_2_O_2_. To the right of each dynamics plot, the initial cell concentration and H_2_O_2_-specific death rate (**b and c**) and initial H_2_O_2_ concentration and cell-specific detoxification rate (**e and f**) are shown.

We follow the same methodology from our *Prochlorococcus* analyses to parameterize the model for *Synechococcus* growing with H_2_O_2_. Figure 6 shows the exponential growth dynamics of *Synechococcus* without H_2_O_2_ addition. Our model was able to adequately capture these dynamics (Fig. 6a), allowing us to quantify the initial cell density and high uncertainty in H_2_O_2_-mediated damage rates (Fig. 6b and c). The model also captured *Synechococcus*-mediated decline in H_2_O_2_ (Fig. 6d), leading to estimates of the initial H_2_O_2_ concentration (Fig. 6e), and the H_2_O_2_ detoxification rate (Fig. 4f). Unlike catalase-deficient *Prochlorococcus*, the catalase-positive *Synechococcus* strain CC9605 grew well in the presence of H_2_O_2_ (Fig. 7a). Using our model, we were also able to capture these different dynamics (Fig. 7a for *Synechococcus* vs Fig. 5a for *Prochlorococcus*), allowing us to quantify the initial cell concentration and the H_2_O_2_-specific rate of cell death, $\kappa_{dam,i}$ (Fig. 7b and c). Similarly, we were also able to capture the dynamics of *Synechococcus* CC9605 cell growth and drawdown of H_2_O_2_ concentration (Fig. 7d), as well as the parameters that represent the initial H_2_O_2_ concentration and *Synechococcus* cell-specific rate of detoxification, $\phi_{det,i}$ (Fig. 7e and f, respectively).

**Fig 8:**
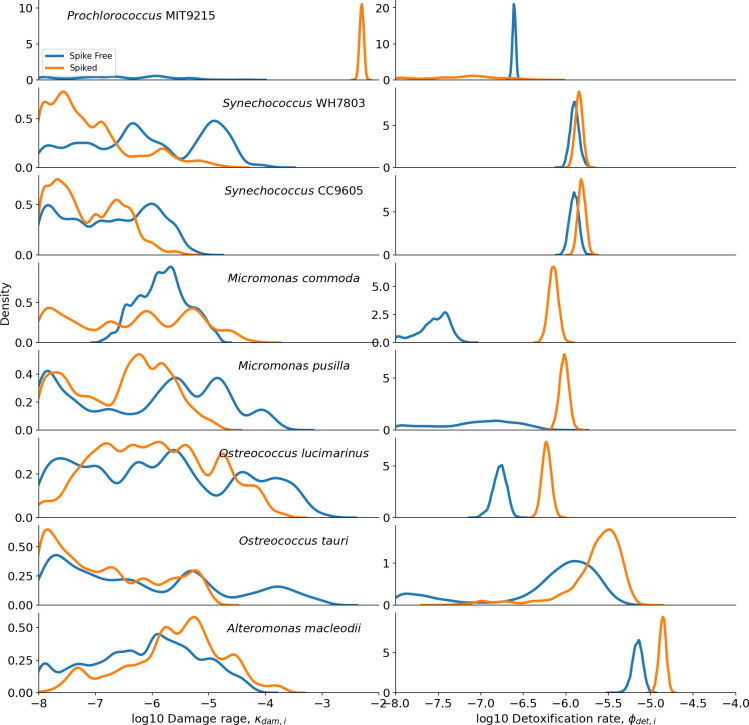
Quantification of uncertainty in H_2_O_2_ trait parameters reveals significant variation between organisms and H_2_O_2_ treatments. Shown are quantitatively recovered estimates of H_2_O_2_-induced rate of cell death (left column; 𝜅_𝑑𝑎𝑚,𝑖⁡_, mL pmol^−1^ day^1^) and H_2_O_2_ detoxification rate (right column; 𝜙_𝑑𝑒𝑡,𝑖_, mL cell^−1^ day^−1^).

After noting the ability of our model to capture the divergent H_2_O_2_ phenotypes of catalase-negative *Prochlorococcus* and catalase-positive *Synechococcus* cyanobacteria, we analyzed further monoculture strains. Additional *Synechococcus* strains, bacterial genera that have previously been isolated with *Prochlorococcus* (33, 63) in the surface ocean, such as *Alteromonas* and picoeukaryotes *Micromonas* and *Ostreococcus*, were analyzed. Results of these additional model-data comparisons are reported in the Supplemental material (Fig. S4 through S18), and a summary of our predictions of H_2_O_2_ damage rates ($\kappa_{dam,i}$) and H_2_O_2_ detoxification rates ($\phi_{det,i}$) for all organisms is shown in Fig. 8. These results reveal significant variation in damage and detoxification rates, both between organisms and by H_2_O_2_ treatment. Importantly, the only damage rate with relatively low uncertainty was *Prochlorococcus* in media spiked with 400 pmol mL^−1^ H_2_O_2_. All other damage rate estimates spanned two or more orders of magnitude (Fig. 8, left column). On the other hand, there was a relatively tight constraint on rates of detoxification. *Prochlorococcus* are capable of detoxification in media unspiked with H_2_O_2_ but are unable to detoxify their environment with the addition of 400 pmol mL^−1^ H_2_O_2_. By contrast, *Synechococcus* rates of detoxification are relatively insensitive to H_2_O_2_ conditions, and the remaining picoeukaryotes and bacteria all appear to upregulate their ability to detoxify ROS in media spiked with 400 pmol mL^−1^ (Fig. 8, right column).

## DISCUSSION

We have presented an ODE-based model of concurrent dynamics of H_2_O_2_ and microbial abundance, which we parameterized using laboratory data. Our model adequately captured H_2_O_2_ dynamics over a range of ecologically relevant H_2_O_2_ concentrations and their differential impacts on closely related cyanobacteria. Our modeling allowed us to quantify two key parameters that describe the impact of H_2_O_2_ on cells ($\kappa_{dam,i}$) and the impact of detoxification on H_2_O_2_ concentration ($\phi_{det,i}$). Using the closely related but genotypically distinct cyanobacteria *Prochlorococcus* and *Synechococcus*, along with known “helper” and catalase-containing heterotroph *Alteromonas*, we demonstrated our model’s ability to capture the duality of divergent H_2_O_2_ detoxification phenotypes. Our ODE-based approach allowed us to quantify parameters in a manner that also accounts for changes in H_2_O_2_ concentration over the course of the experiment. Because many marine ecosystem models (69) are also based on differential equations, the parameters we have identified ($\kappa_{dam,i}$ and $\phi_{det,i}$) can be used to evaluate the impacts of H_2_O_2_ on microbial community composition and carbon cycling within these more complex models.

Our model was compared with dynamics of marine microbes, but it may be applicable to organisms growing in a wide range of habitats. Catalase has been discovered in all domains of life (70) including most cultured, aerobic microbial species (16, 71, 72). Despite being found across the tree of life, catalases are found infrequently in natural samples (16). In contrast, monofunctional peroxidases are found across the tree of life and are also widespread, including within *Prochlorococcus*, despite not being the most efficient enzyme capable of detoxifying H_2_O_2_. The widespread pervasiveness of peroxidases and relative rarity of catalases suggest there are costs and benefits to ROS detoxification that may be common to a wide range of environmental conditions. Our model is a step toward quantitative exploration of ROS-mediated competitive interactions and may be applicable beyond the marine environment, which was the focus of the present study.

A limitation of our study is that the laboratory conditions do not fully replicate conditions cells experience in the open ocean. For example, the modeled experiments introduced single spikes of H_2_O_2_ which, while consistent with rain inputs for surface waters (36, 73), are different from the continual H_2_O_2_ photoproduction in natural systems that follow a diel cycle associated with changes in light and DOC concentration (34, 61, 74). Use of chemical buffers to control H_2_O_2_ production (61) is one way to more closely mimic natural H_2_O_2_ production, and could be accounted for via alterations of our H_2_O_2_ production parameter, S_H_. To constrain our model with experimental data, we require time-dependent measurements of both H_2_O_2_ and cell abundance, which were only available in the spike assay data used here. Fitting our model to experiments with chemical buffer would provide a more realistic and general test of this framework. But we do not anticipate this would fundamentally change any of our main findings regarding detoxification and cell death parameters.

An additional limitation of our study is that the ODEs in our model account for changing cell density but assume a fixed cell state; i.e., we do not account for changing transcription levels of detoxification genes that occur in microbial populations in response to increased reactive oxygen or other environmental pressures (72, 75, 76). Furthermore, the batch culture conditions used for our laboratory cultures are not reflective of the nutrient-limiting conditions prevalent in the oligotrophic systems where *Prochlorococcus* thrives. Applying our model to *Prochlorococcus* growing in chemostat conditions—where nutrients are more likely to be limiting—under a range of nitrogen inputs would be a powerful additional test of its applicability. A further test of our model would be to compare it with data from experiments of microbes grown under contrasting light intensities and temperature conditions (42). While these considerations are exciting future extensions of our model-data comparisons, we do not anticipate that the analysis would qualitatively modify the main findings reported here, that catalase-positive bacteria and picoeukaryotes tend to have far higher rates of cell-specific detoxification than *Prochlorococcus* and have significantly diminished rates of cell death associated with H_2_O_2_ damage.

A powerful additional test of our model would be to ask whether it can predict the outcome of experiments with one or more organisms growing in coculture. However, organisms growing in coculture interact in many ways, e.g., exchanging reduced carbon and nitrogen (77), competing for nutrients (78), and exchanging infochemicals (79). Our model provides a basis on which to explore the mechanisms driving these interdependencies now and in future oceans, but incorporation of these mechanisms would go beyond the scope of the current study. A related issue is the continued excretion of DOC by *Prochlorococcus* (80–83), which may augment H_2_O_2_ production from light (34) and other sources. Our model did not explicitly resolve the sensitivity of H_2_O_2_ production to DOC concentration, but its impact is implicitly accounted for in our quantification of H_2_O_2_-mediated *Prochlorococcus* cell death. Nevertheless, DOC-mediated production of H_2_O_2_ is likely to be an important process in any environmentally relevant model of H_2_O_2_ dynamics, especially in situations where DOC chemical composition and associated photochemical H_2_O_2_ production vary in time and space. Further analysis of H_2_O_2_ production in spike assays (84) and chemical buffers or natural DOC [e.g., HEPES, 4-(2-hydroxyethyl)piperazine-1-ethane-sulfonic acid (19, 61)] may elucidate and constrain differences between laboratory and ocean observations.

Despite its limitations, our ODE framework provides a basis for incorporation of H_2_O_2_-mediated competition into biogeochemical models. The goal of such an effort would be to ask whether H_2_O_2_ influences microbial community composition and mortality. A major step toward this goal would be to incorporate a representation of H_2_O_2_ production and decay within an ocean context. A representation of H_2_O_2_ production would need to incorporate photochemical production, rain, and cellular sources of H_2_O_2_, as well as conversions with other ROSs, such as hydroxyl radicals and superoxide. Photochemical production and cellular-mediated production can each produce enough H_2_O_2_ to kill *Prochlorococcus* (19, 23, 34), and their combined impact on community dynamics, along with rain, has unknown impacts on microbial ecosystem structure and function. Climate change is impacting ocean carbon and nitrogen fluxes (85–88) while altering marine temperature (89) and precipitation (12) inputs, all of which either directly or indirectly affect H_2_O_2_ concentrations (31, 32, 35, 90, 91). We anticipate that quantification of H_2_O_2_ detoxification and susceptibility in multiple catalase-positive and catalase-negative plankton groups will help future analyses exploring the impacts of multiple stressors oligotrophic microbial communities experience in a changing world.

## Data Availability

Computer codes are available at https://github.com/DKMcCullough/HOOH_dynamics, and data are available in reference 59.

## SUPPLEMENTAL MATERIAL

Supplemental material spectrum.00712-25-s0001.docx Supplemental methods; Fig. S1 to S18.

ASM does not own the copyrights to Supplemental Material that may be linked to, or accessed through, an article. The authors have granted ASM a non-exclusive, world-wide license to publish the Supplemental Material files. Please contact the corresponding author directly for reuse.
