# Supplementary material for: Quantification of the coupled dynamics of marine microbes and reactive oxygen species in laboratory batch culture experiments
Source: Microbiol Spectr. 2026 May 12;14(6):e00712-25. doi: 10.1128/spectrum.00712-25 (PMC13228047; doi:10.1128/spectrum.00712-25)
Supplement: Supplemental material — Supplemental methods; Fig. S1 to S18. [file spectrum.00712-25-s0001.docx]

**Supplementary Information for:** Quantification of the coupled dynamics of marine microbes and reactive oxygen species in laboratory batch culture experiments

**Authors:** Donna K. McCullough^1^, Emily Bowden^1^, Benjamin C. Calfee^1^, Michael A. Gilchrist^2^, Erik Zinser^1^, David Talmy^1,*^

^1^Department of Microbiology, University of Tennessee, Knoxville, TN, USA

^2^Department of Ecology and Evolutionary Biology, University of Tennessee, Knoxville, TN, USA

^*^Corresponding author: [dtalmy@utk.edu](mailto:dtalmy@utk.edu)

## **S.I. 1 Parameter fitting procedure**

The goal of our fitting procedure was to search for probability distributions that maximize the probability of each parameter being true, given the model and the experimental data. Let a given experimental dataset be labelled *D*, and a set of parameters be $\theta$. In the following, we describe a procedure to estimate the probability of the parameters given the observations, which may be written $P(\theta$ |D). The following is true for any specific choice of data (e.g. *Prochlorococcus* cell densities through time, main text Figure 4a) and corresponding parameter set (e.g. $\theta=\{P_{i,0},\mu_{max}$}, main text Figure 4b,c). Using Bayes theorem, the probability of a given parameter set given the observations is:

(eq S1 ) $P(\theta|D) =\frac{P( D|\theta)P(\theta)}{P(D)}$

In practice, it is difficult to directly find the set $\theta$ which agrees with the probability in Equation S1. The Metropolis-Hastings algorithm approximates the set $\theta$ with a random walk procedure. Consider first assuming a given parameter set, which we call $\theta_{old}$. The essence of the random walk algorithm is to consider the feasibility of this parameter set by comparing it to a competing set of parameters, which we call $\theta_{proposed}$. By Equation S1, the ratio of $P(\theta_{proposed}|D)$ to $P(\theta_{old}|D)$ may be written

(eq S2) $\frac{P\left( \theta_{proposed} | D \right)}{P\left( \theta_{old} | D \right)}=\frac{P\left( D | \theta_{proposed} \right)}{P\left( D | \theta_{old} \right)}\frac{P\left( \theta_{proposed} \right)}{P\left( \theta_{old} \right)}$

Note that $P(D)$ does not appear in Equation S2, which is helpful as this quantity is difficult to ascertain empirically. Equation S2 requires knowledge of $P( D|\theta)$, the probability of the data for a given parameter set, and $P(\theta)$, the probability of a given parameter set independent of anything else. Before defining these mathematically, we focus attention on procedural aspects of the Metropolis-Hastings algorithm.

The Metropolis-Hastings algorithm generates each new parameter set following a random walk procedure:

(eq S3) $\mu_{prop}=\mu_{old}e^{a}$ $\tau_{prop}=\tau_{old}e^{b}$ $\varphi_{prop}=\varphi_{old}e^{c}$ $\varphi_{prop}=\varphi_{old}e^{d}$

where *a*, *b*, *c* and *d* are independent random numbers drawn from a normal probability distribution with mean zero and standard deviation 0.05. The precise choice of standard deviation modifies the computational efficiency of the algorithm but should not change the results (Robert and Casella 2010). If the new parameter set is more probable than the old parameter set, then the ratio of $P(\theta_{proposed}|D)$ to $P(\theta_{old}|D)$ (Equation S2) will be greater than one. If this is the case, then we can adopt this parameter set by relabeling it $\theta_{new}$. We then generate another $\theta_{proposed}$ with Equation 7 (this time $\theta_{new}$ becomes $\theta_{old})$, and repeat this procedure iteratively until the random walk navigates to the set that best describes the probability in Equation S1. A subtle but key additional feature of the Metropolis-Hastings algorithm is to search parameter space by occasionally letting worse parameter sets be adopted. It does this by accepting the new parameter set if:

(eq S4) $\frac{P\left( \theta_{proposed} | D \right)}{P\left( \theta_{old} | D \right)}e^{a+b+c+d}\geq r$

Where *r* is drawn randomly from a uniform distribution within the range [0,1]. Note that the exponential term in Equation S4 helps the algorithm compensate for the asymmetry of the lognormal distribution, and its tendency to favor large values over small values. If the ratio in Equation S4 is greater than *r*, then it must also be greater than one, and hence improvements to the parameter set are always accepted. However, every so often, the ratio in Equation S4 will be less than one but greater than *r*. Here, poorer parameter sets are still opted for. The algorithm still converges upon optimal parameter sets since there is a stronger tendency to select improved parameter sets over poorer ones.

We now return to the unknowns in Equation S2: the probability of the data being true with respect to a given parameter set (i.e. $P( D|\theta)$), and the probability of each parameter set being true, independent of anything else (i.e. $P(\theta)$). In practice, these are typically quantified by a likelihood, and a set of priors, respectively. The likelihood of the data being true for any proposed parameter set is calculated with a likelihood function, $\mathcal{L(}\theta)$, which is defined as follows:

(eq S5) $P\left( D | \theta\right) =\mathcal{L(}\theta)=exp\left( {-\chi}^{2} \right)$

Where $\chi^{2}$ is the squared sum of differences between log-transformed hydrogen peroxide and cell abundances and the corresponding model estimate, evaluated at $\theta$ (labelled here $y_{i,m}$):

(eq S6) $\chi^{2}=\sum_{i} \frac{\left( \bar{\ln\left( y_{i,j} \right)}-ln(y_{i,m}) \right)^{2}}{\sigma_{y,i}}$

In Equation S6, $y_{i,j}$ represents the population size at time-point *i=1,n*, and replicate *j=1,m*, and $\bar{\ln\left( y_{i,j} \right)}$ is the average of replicates after log-transformation. The parameter $\sigma_{y,i}$ is an uncertainty value, which sets the confidence with which we believe each datapoint is true. The specific choice of $\sigma_{y,i}$ was estimated for each dataset separately by analyzing the standard deviation among replicates or triplicates of log-transformed hydrogen peroxide and cell abundance data (see Section S.I. 2 and Figures S1-S3).

The probability of a given parameter set being true, ($P(\theta)$ in Equation S5), is set by priors, which we have the freedom to choose. We assumed uninformative priors by neglecting $P(\theta)$, allowing all parameter values to be equally feasible before the fitting procedure. For each model-data fit, the random walk was run for 100,000 steps. The initial 50,000 iterations of the random walk were discarded. In most cases, posterior probability distributions did not change when simulations were run for different timestep lengths, indicating that the parameters random walks reliably converged upon well determined values.

##

## **S.I. 2 Quantifying uncertainty in experimental data**

The Metropolis-Hastings algorithm takes information about the uncertainty of each experimental datapoint to quantify parameter posterior distributions. Our likelihood function (Equations S5 and S6) assumes log-transformed data are normally distributed. This assumption was based on our observation that strong correlation between standard deviation and mean of biological replicates (Figure S2.1, left column) largely disappeared after log-transformation (Figure S1, right column). Strong positive correlation between standard deviation and mean is an indication that a multiplicative process generates variance in biological replicates. The lack of clear structure in the relationship between standard deviation and mean after log-transformation suggests a log-normal distribution may be an appropriate description of variance. The log-normal distribution is widely used to model variance in biological systems for theoretical and empirical reasons (Koch 1966; Dennis et al. 1987; Limpert et al. 2001).

| 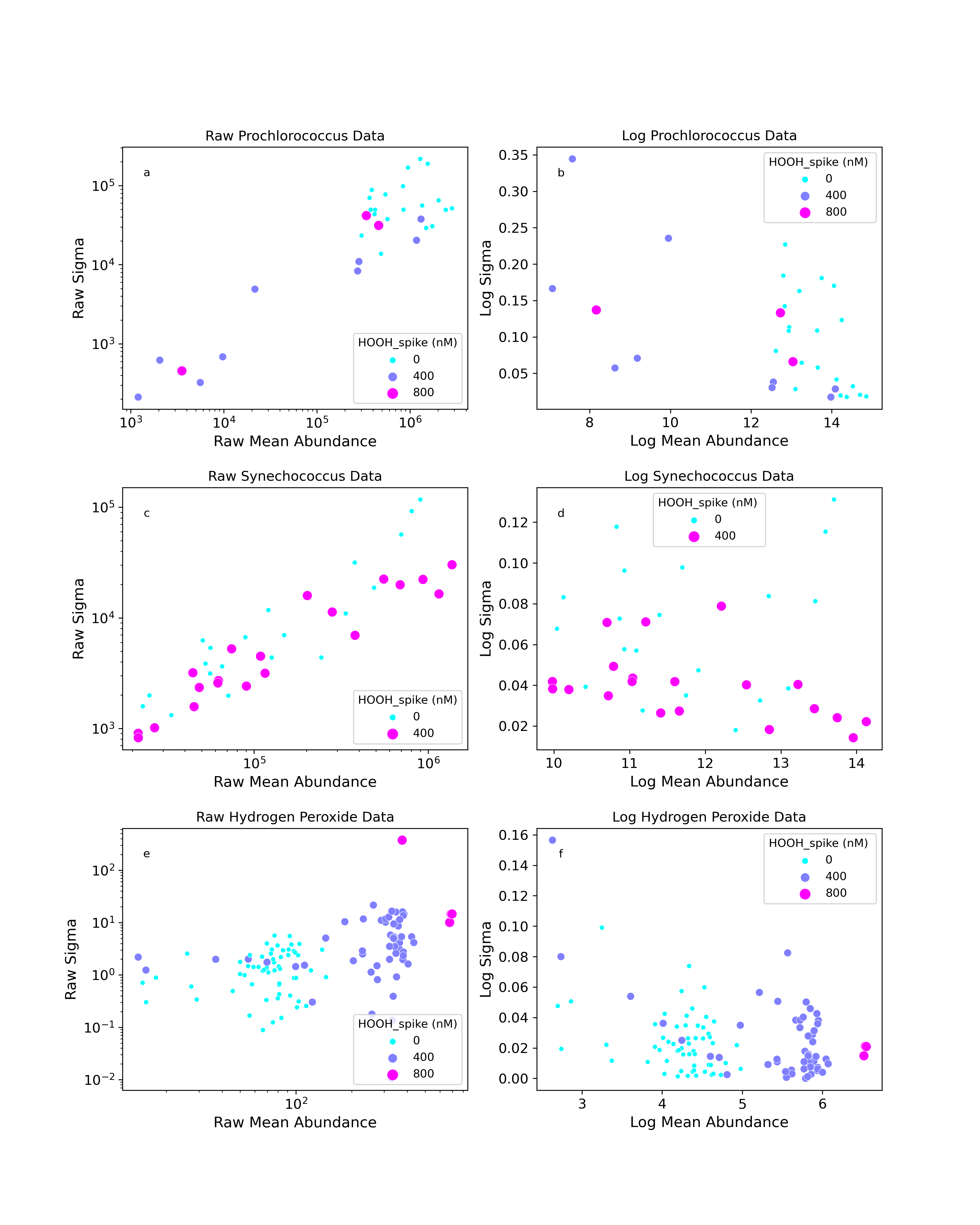 |
| --- |
| **Figure S1:** Analysis of the relationship between standard deviation and mean among biological triplicates for raw (left column) vs log-transformed (right column) a,b) *Prochlorococcus* abundance data c,d) *Synechococcus* abundance data and e,f) Hydrogen peroxide concentrations. There is greater positive correlation between mean and standard deviations in untransformed data, which is largely removed after log-transformation. We interpret this as evidence of multiplicative noise-generating processes, and assume log-transformed data are normally distributed. |

We first assumed that the uncertainty parameter, $\sigma_{y,i}$ could be constrained by the standard deviation among biological replicates or triplicates:

(eq S7) $\sigma_{y,i}^{2}=\sum_{j} \left( \ln\left( y_{i,j} \right)-\bar{\ln\left( y_{i,j} \right)} \right)^{2}$

Where $y_{i,j}$ represents the population size at time-point *i=1,n*, and replicate *j=1,m*, and $\bar{\ln\left( y_{i,j} \right)}$ is the average of replicates. Within each dataset, we initially constructed a 99% confidence interval on the uncertainty parameter, by assuming that the standard deviation follows a chi-squared distribution (Sheskin 2003):

(eq S8) ${\sqrt{\frac{m-1}{\chi_{\frac{\alpha}{2},m-1}^{2}}}<\sigma}_{y,i}<\sqrt{\frac{m-1}{\chi_{1-\frac{\alpha}{2},m-1}^{2}}}$

Where $\alpha$ is the desired confidence level which in our case is 1-0.99=0.01. Due to the high-level of uncertainty associated with sample size *m*=2 or *m*=3 (as is the case for biological replicates or triplicates, respectively), Equation S8 led to unrealistically wide confidence intervals for the standard deviations. We therefore estimated the standard deviation across time-points. To do this, we first subtracted the mean of log-transformed replicates from each log-transformed replicate (i.e. $\tilde{y}_{i,j}=\ln\left( y_{i,j} \right)-\bar{\ln\left( y_{i,j} \right)}$ ). We assumed that each $\tilde{y}_{i,j}$ was normally distributed with mean 0 and unknown variance $\sigma_{y}$ which we assumed was equal across time-points (i.e. $\sigma_{y}=\sigma_{y,i}$ for all i=1,n). We then calculated the variance of $\tilde{y}_{i,j}$ and used Equation S9 to again construct 99% confidence intervals, this time however, with *n-1* degrees of freedom:

(eq S9) ${\sqrt{\frac{n-1}{\chi_{\frac{\alpha}{2},n-1}^{2}}}<\sigma}_{y}<\sqrt{\frac{n-1}{\chi_{1-\frac{\alpha}{2},n-1}^{2}}}$

In Figure S2.2a, we show hydrogen peroxide replicates in media with zero H_2_O_2_ addition. The blue dots in Figure S.2 2b show log-transformed standard deviations among replicates (i.e. $\sigma_{y,i}$). The red-dashed line is the average standard deviation across time-points, and the solid red line is the upper limit of the 99% confidence interval in Equation S9. The solid red line was used to constrain $\sigma_{y,i}$ in Equation S6. A different $\sigma_{y,i}$ was calculated for each dataset *D* following the same procedure. An example of this for *Synechococcus* cell dynamics is shown in Figure S2.3.

| 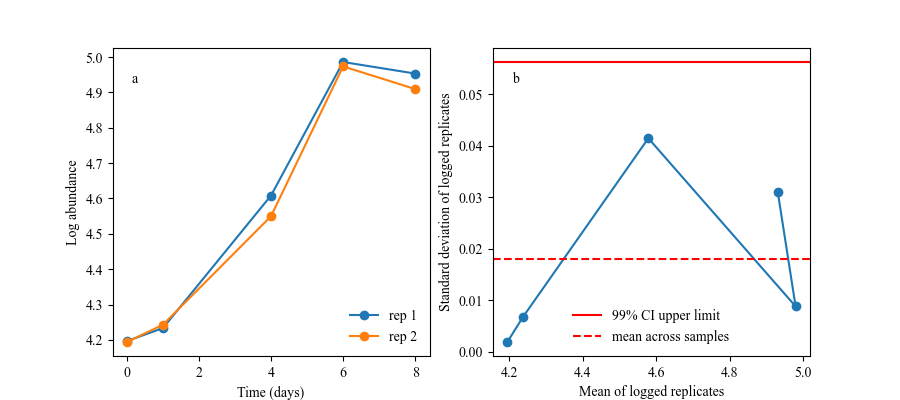 |
| --- |
| **Figure S2:** replicates of hydrogen peroxide concentration (a) along with a depiction of different quantities to constrain uncertainty across time-points (b). The red dashed line marked by the upper limit of a 99% confidence interval was determined with Equation 10 and set the uncertainty parameter $\sigma_{y,i}$ across timepoints in this dataset. |

| 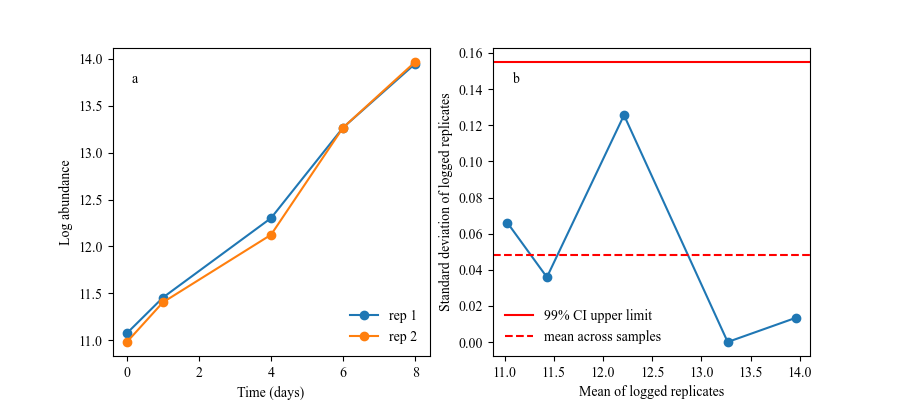 |
| --- |
| **Figure S3:** replicates of *Synechococcus* abundance (a) along with a depiction of different quantities to constrain uncertainty across time-points (b). All lines and symbols have the same meaning as in Figure S2. |

### **S.I.3 Model fits to laboratory cell and H_2_O_2_ dynamics for diverse microbes**

| 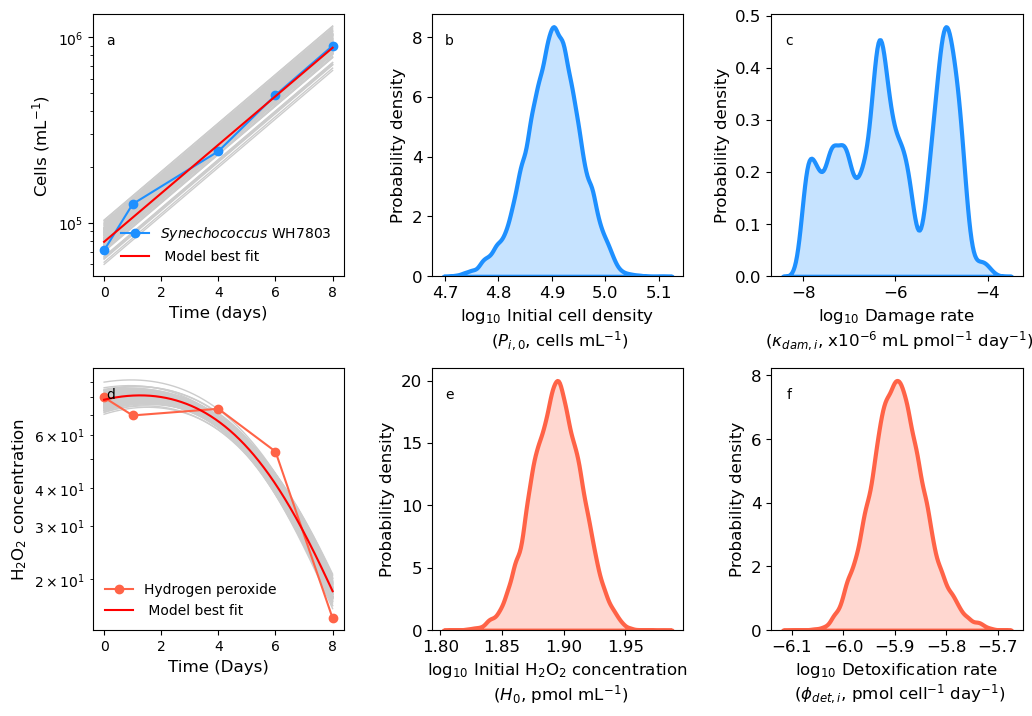 |
| --- |
| **Figure S4:** Coupled laboratory batch culture dynamics of Synechococcus WH7803 (a) and H_2_O_2_ (d) data alongside model best-fit (red line) and searched (grey) combinations in laboratory assays containing no added hydrogen peroxide. To the right of each dynamics plot the initial value of Synechococcus WH7803, P_i,0_ (b) and hydrogen peroxide, H_0_ (e) and the controlling parameter for each line: damage rate κ_dam,i_ (c) and detoxification rate ϕ_det,i_ H (e) are shown. |

| 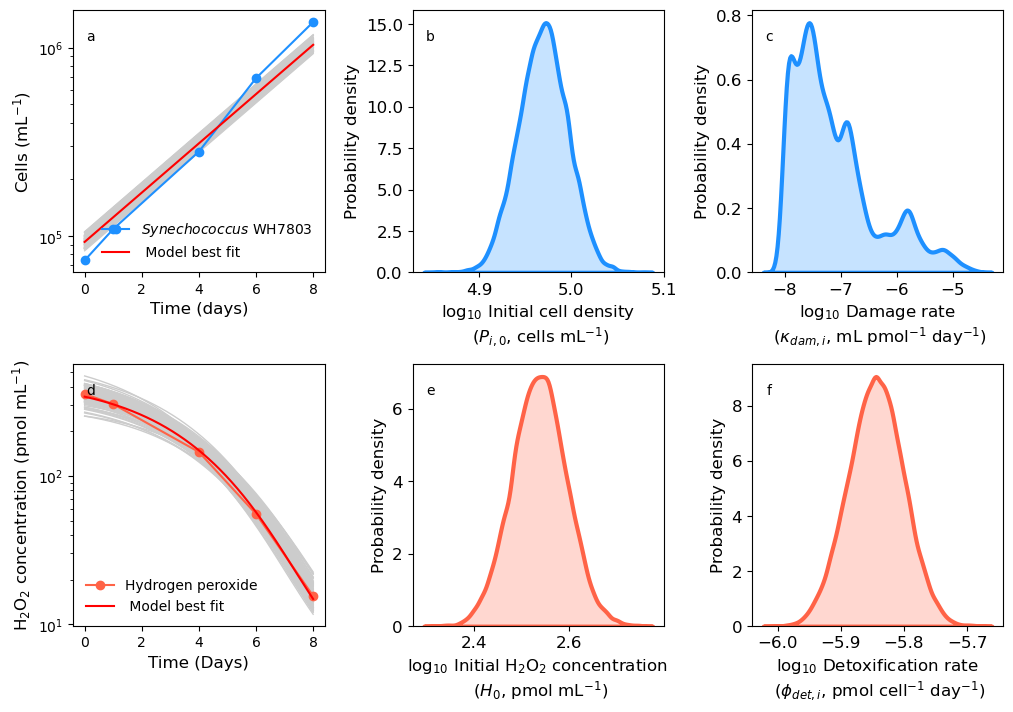 |
| --- |
| **Figure S5:** Coupled laboratory batch culture dynamics of *Synechococcus* WH7803 (a) and H_2_O_2_ (d) data alongside model best-fit (red line) and searched (grey) combinations in laboratory assays containing a 400 pmol mL^-1^ H_2_O_2_ spike. To the right of each dynamics plot the initial value of *Synechococcus* WH7803, P_i,0_ (b) and hydrogen peroxide, H_0_ (e) and the controlling parameter for each line: damage rate κ_dam,i_ (c) and detoxification rate ϕ_det,i_ (f) are shown. |

| 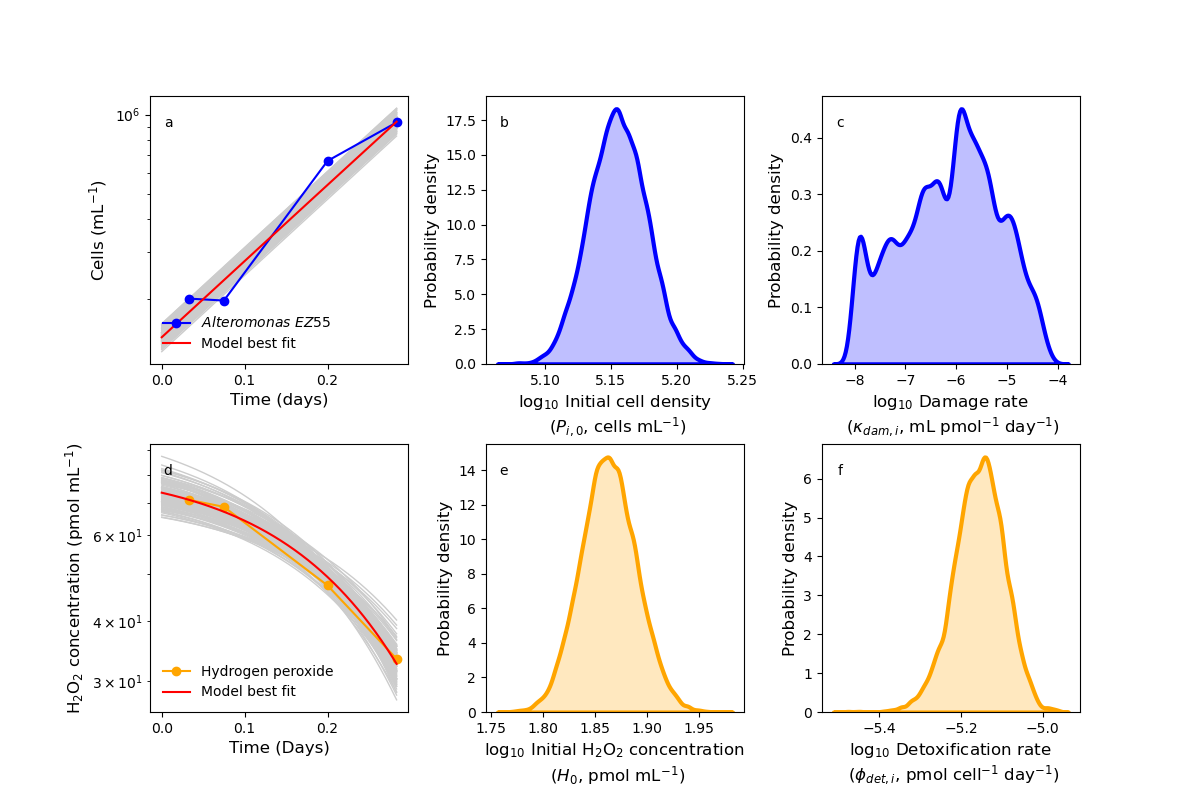 |
| --- |
| **Figure S6:**  Coupled laboratory batch culture dynamics of Alteromonas EZ55 (a) and H_2_O_2_ (d) data alongside model best-fit (red line) and searched (grey) combinations in laboratory assays containing no added hydrogen peroxide. To the right of each dynamics plot the initial value of Alteromonas EZ55, P_i,0_ (b) and hydrogen peroxide, H_0_ (e) and the controlling parameter for each line: damage rate κ_dam,i_ (c) and detoxification rate ϕ_det,i_ (f) are shown. |

| 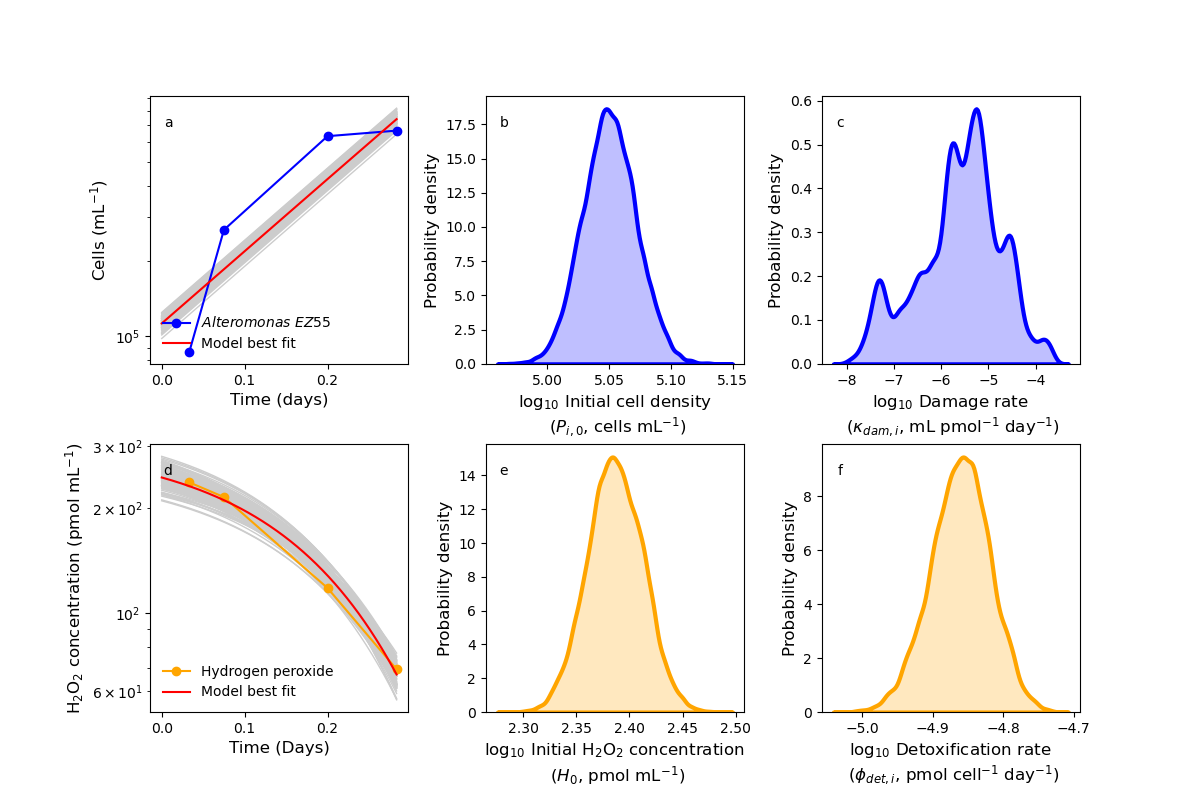 |
| --- |
| **Figure S7:** Coupled laboratory batch culture dynamics of *Alteromonas* EZ55 (a) and H_2_O_2_ (d) data alongside model best-fit (red line) and searched (grey) combinations in laboratory assays containing a 400 pmol mL^-1^ H_2_O_2_ spike. To the right of each dynamics plot the initial value of *Alteromonas* EZ55, P_i,0_ (b) and hydrogen peroxide, H_0_ (e) and the controlling parameter for each line: damage rate κ_dam,i_ (c) and detoxification rate ϕ_det,i_ (f) are shown. |

| 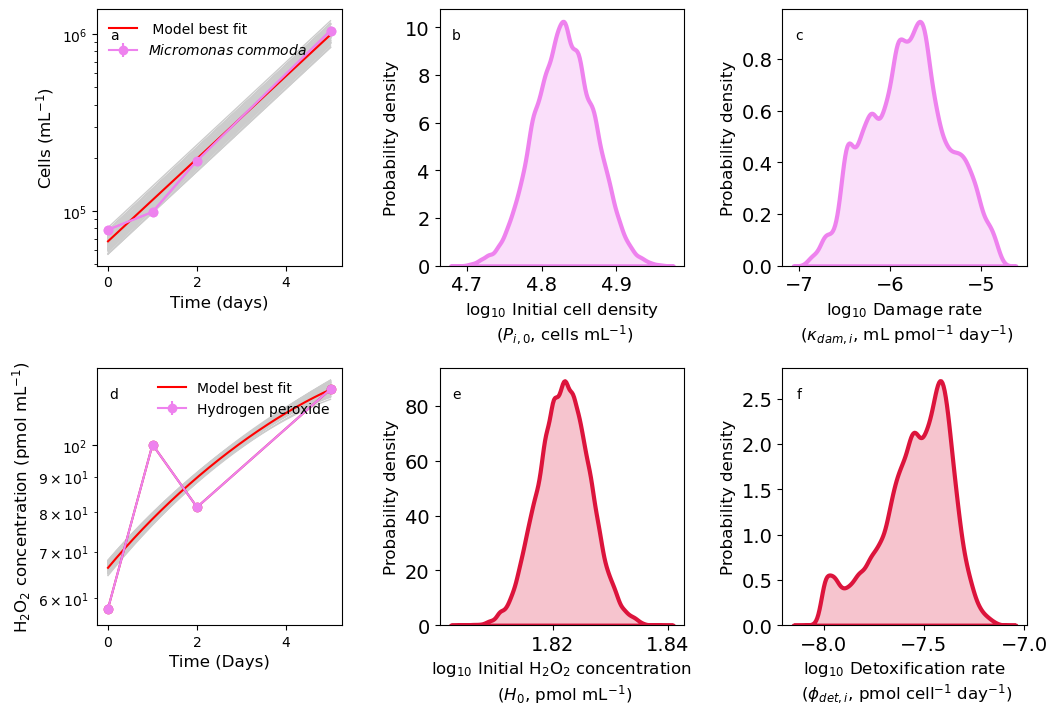 |
| --- |
| **Figure S8:** Coupled laboratory batch culture dynamics of Micromonas commoda (a) and H_2_O_2_ (d) data alongside model best-fit (red line) and searched (grey) combinations in laboratory assays containing no added hydrogen peroxide. To the right of each dynamics plot the initial value of Micromonas commoda , P_i,0_ (b) and hydrogen peroxide, H_0_ (e) and the controlling parameter for each line: damage rate κ_dam,i_ (c) and detoxification rate ϕ_det,i_ (f) are shown. |

|  |
| --- |
| **Figure S9:** Coupled laboratory batch culture dynamics of the detoxifier *Micromonas commoda* (pink line) (a) and H_2_O_2_ (maroon line) (d) data alongside searched parameter combinations (grey) in batch culture containing 400 pmol mL^-1^ H_2_O_2_ spike. To the right of each dynamics plot we show the initial cell concentration and H_2_O_2_ specific death rate (b,c) and initial hydrogen peroxide concentration and cell-specific detoxification rate (e,f). |

| 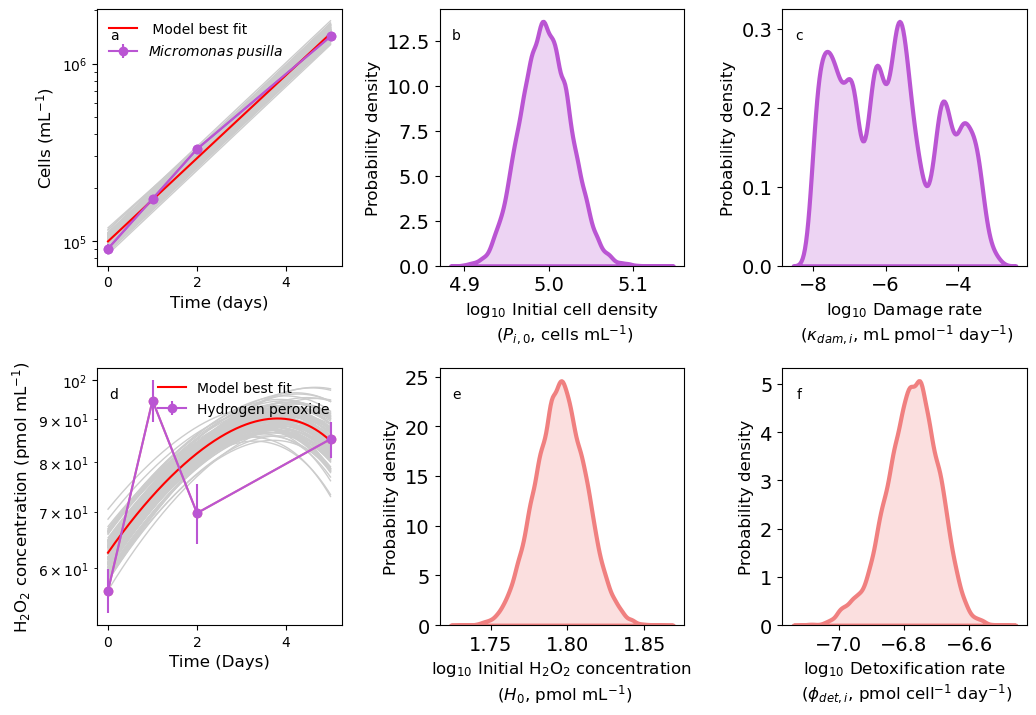 |
| --- |
| **Figure S10:** Coupled laboratory batch culture dynamics of Micromonas pusilla (a) and H_2_O_2_ (d) data alongside model best-fit (red line) and searched (grey) combinations in laboratory assays containing no added hydrogen peroxide. To the right of each dynamics plot the initial value of Micromonas pusilla, P_i,0_ (b) and hydrogen peroxide, H_0_ (e) and the controlling parameter for each line: damage rate κ_dam,i_ (c) and detoxification rate ϕ_det,i_ (f) are shown. |

| 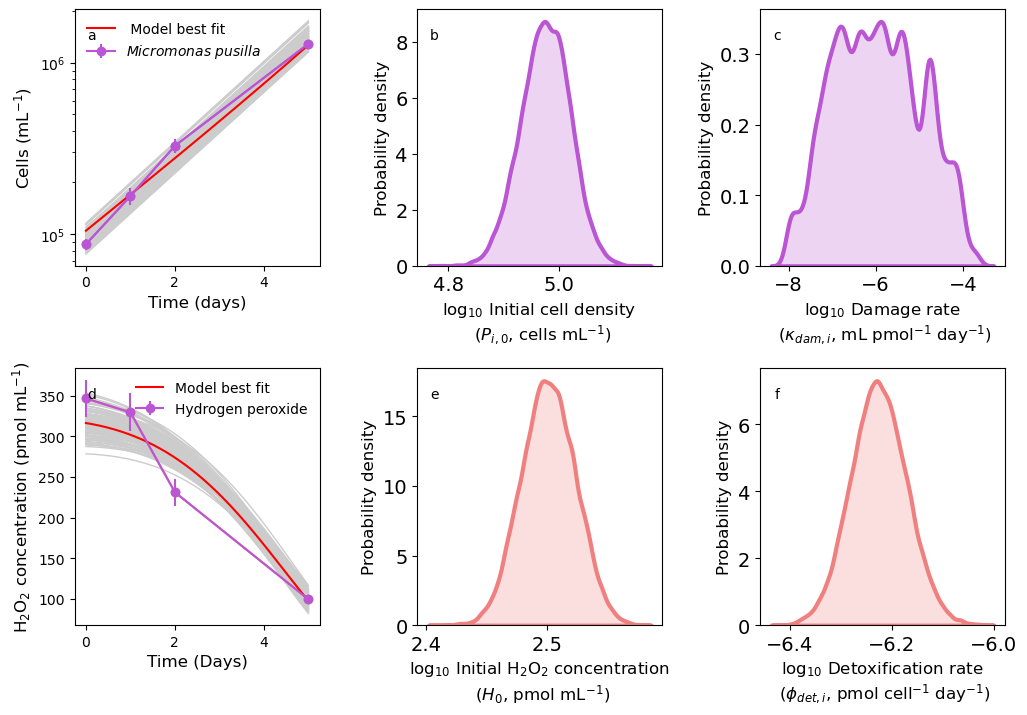 |
| --- |
| **Figure S11:** Coupled laboratory batch culture dynamics of the detoxifier Micromonas pusilla (purple line) (a) and H_2_O_2_ (pink line) (d) data alongside searched parameter combinations (grey) in batch culture containing 400 pmol mL^-1^ H_2_O_2_ spike. To the right of each dynamics plot we show the initial cell concentration and H_2_O_2_ specific death rate (b,c) and initial hydrogen peroxide concentration and cell-specific detoxification rate (e,f). |

| 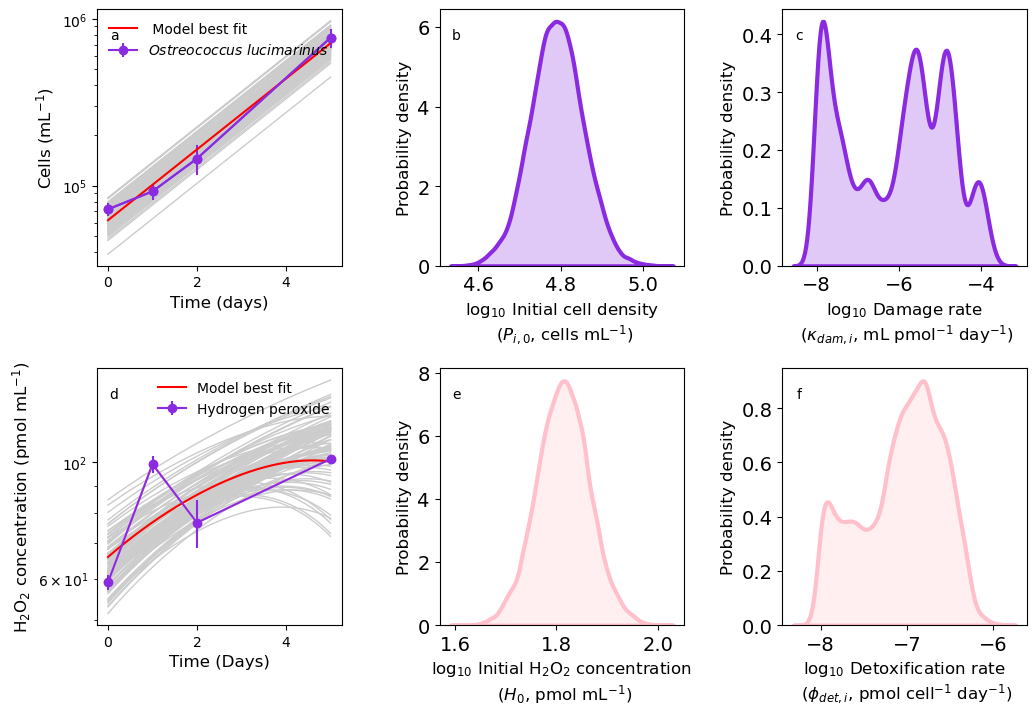 |
| --- |
| **Figure S12:** Coupled laboratory batch culture dynamics of Ostreococcus lucimarinus (a) and H_2_O_2_ (d) data alongside model best-fit (red line) and searched (grey) combinations in laboratory assays containing no added hydrogen peroxide. To the right of each dynamics plot the initial value of Ostreococcus lucimarinus, P_i,0_ (b) and hydrogen peroxide, H_0_ (e) and the controlling parameter for each line: damage rate κ_dam,i_ (c) and detoxification rate ϕ_det,i_ (f) are shown. |

| 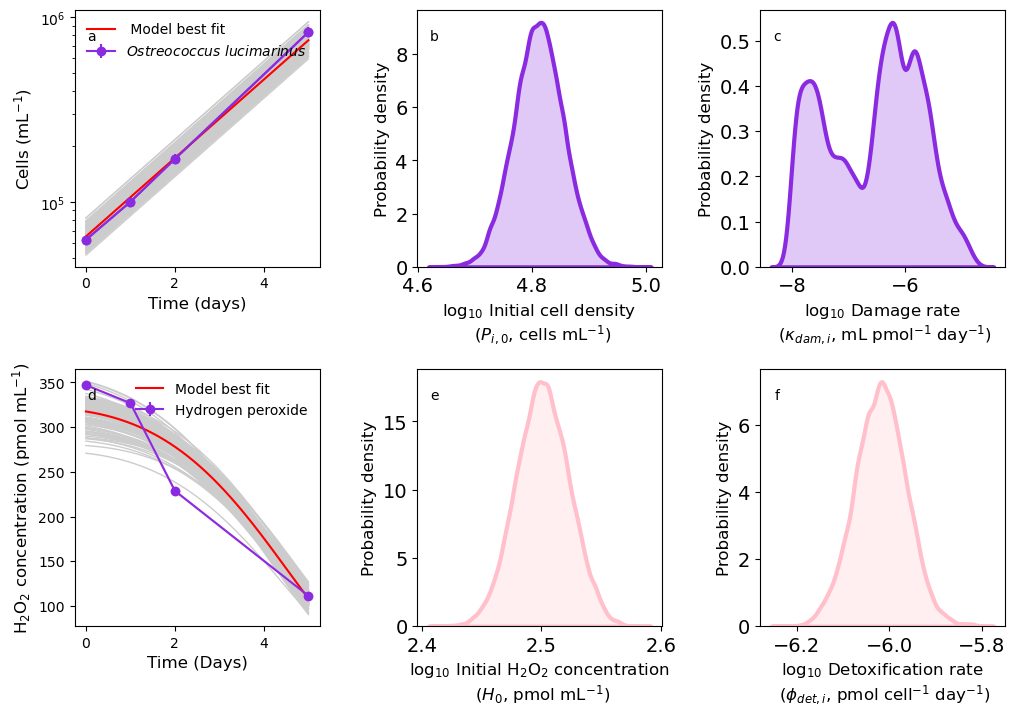 |
| --- |
| **Figure S12:** Coupled laboratory batch culture dynamics of Ostreococcus lucimarinus (a) and H_2_O_2_ (d) data alongside model best-fit (red line) and searched (grey) combinations in laboratory assays containing no added hydrogen peroxide. To the right of each dynamics plot the initial value of Ostreococcus lucimarinus, P_i,0_ (b) and hydrogen peroxide, H_0_ (e) and the controlling parameter for each line: damage rate κ_dam,i_ (c) and detoxification rate ϕ_det,i_ (f) are shown. |

| 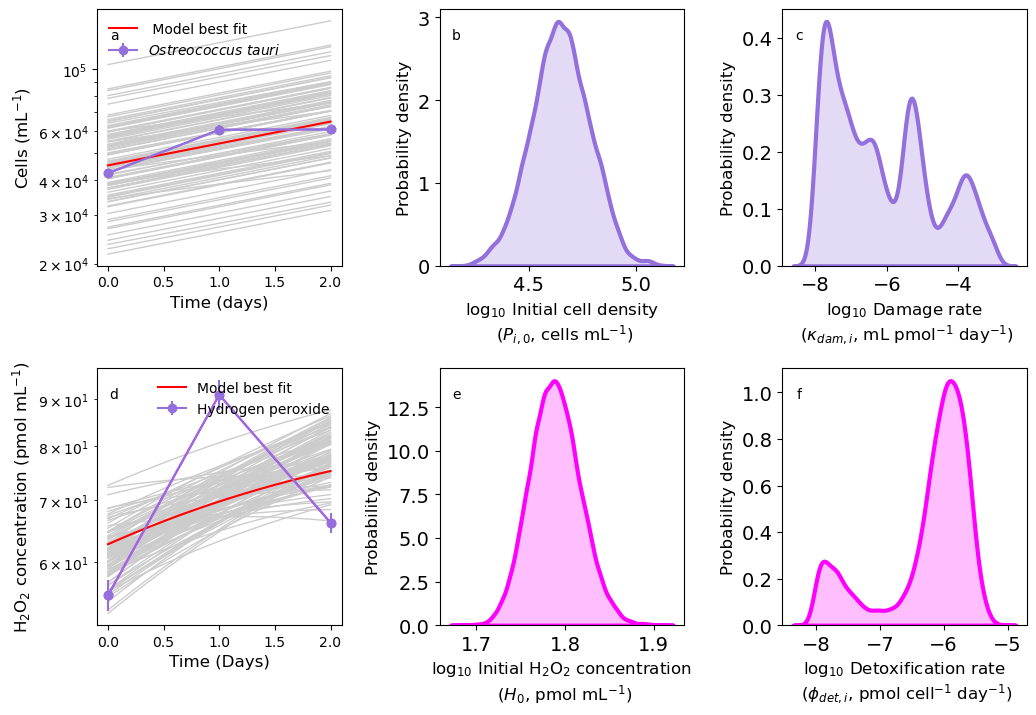 |
| --- |
| **Figure S14:** Laboratory batch culture dynamics of the detoxifier Ostreococcus tauri with data (purple line) and model searches (grey lines) with optimal solution (red line) in zero H_2_O_2_ batch culture (a). Also shown are parameter value histograms for the initial cell concentration and exponential growth rate (b,c, respectively). |

| 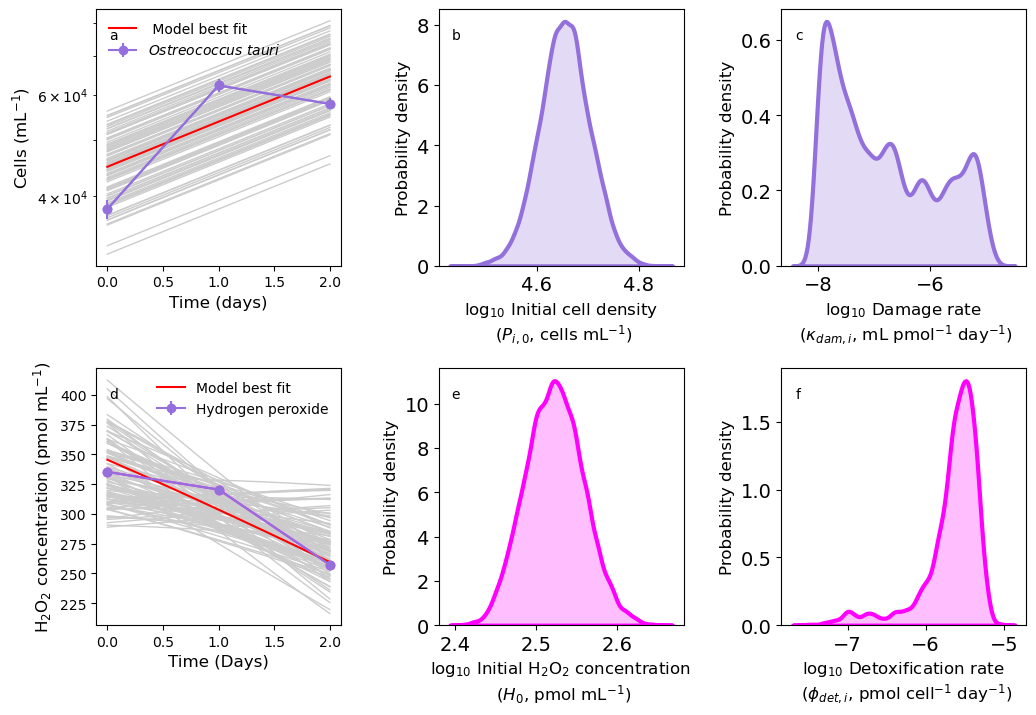 |
| --- |
| **Figure S15:** Coupled laboratory batch culture dynamics of the detoxifier Ostreococcus tauri (a) and H_2_O_2_ (d) data alongside searched parameter combinations (grey) in batch culture containing 400nM H_2_O_2_. To the right of each dynamics plot we show the initial cell concentration and H_2_O_2_ specific death rate (b,c) and initial hydrogen peroxide concentration and cell-specific detoxification rate (e,f). |

| 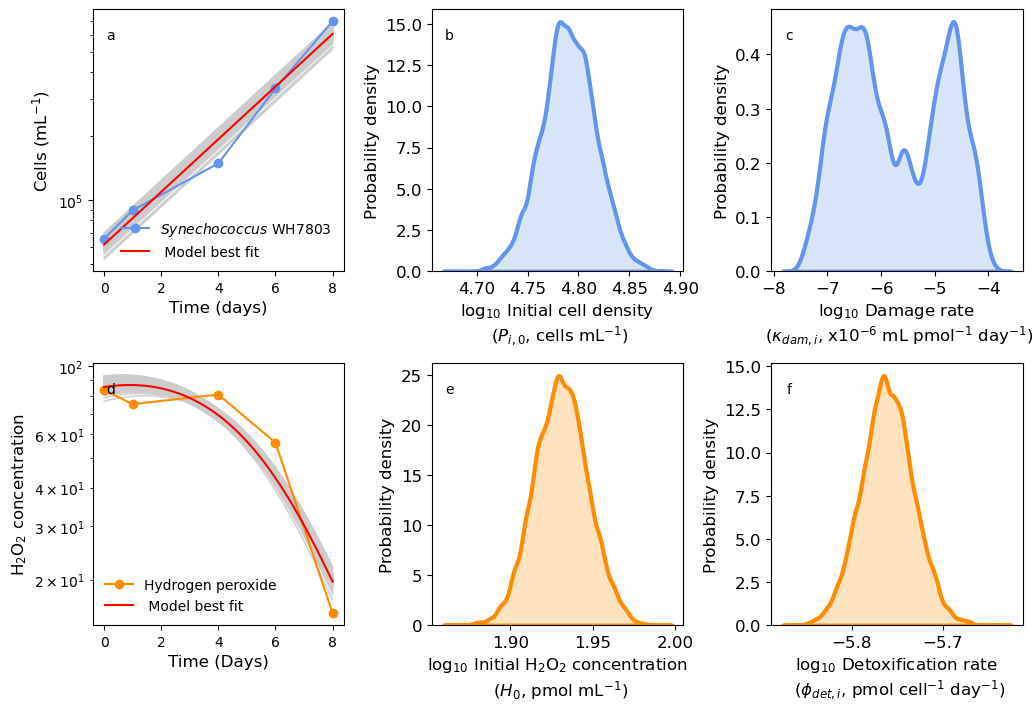 |
| --- |
| **Figure S16:** Laboratory batch culture dynamics of Synechococcus WH7803 (Vol #28) and model searches (grey lines) with optimal solution (red line) in zero H_2_O_2_ batch culture (a). Also shown are parameter value histograms for the initial cell concentration and exponential growth rate (b,c, respectively). |

| 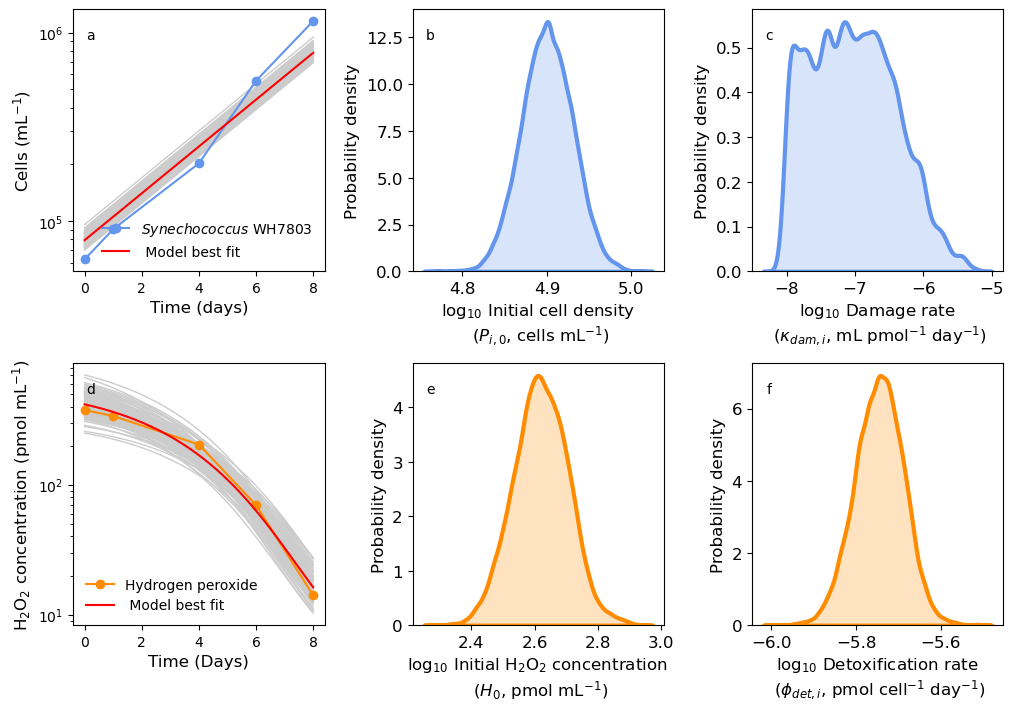 |
| --- |
| **Figure S17:** Coupled laboratory batch culture dynamics of *Synechococcus* WH7803 (Vol# 28) (a) and H_2_O_2_ (d) alongside searched parameter combinations (grey) in batch culture containing a 400 pmol mL^-1^ H_2_O_2_ spike. To the right of each dynamics plot we show the initial cell concentration and H_2_O_2_ specific death rate (b,c) and initial hydrogen peroxide concentration and cell-specific detoxification rate (e,f). |

| 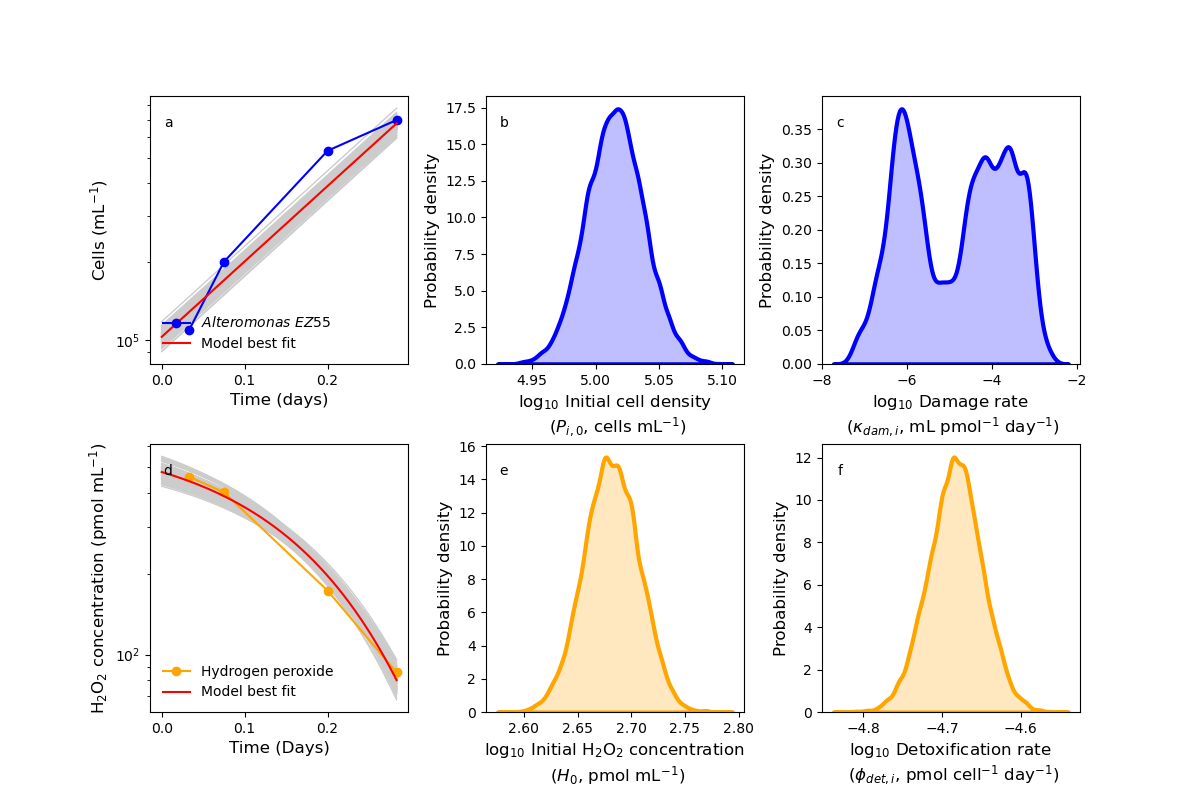 |
| --- |
| **Figure S18:** Coupled laboratory batch culture dynamics of Alteromonas EZ55 (a) and H_2_O_2_ (d) data alongside searched parameter combinations (grey) in batch culture containing a 800 pmol mL^-1^ H_2_O_2_ spike. To the right of each dynamics plot we show the initial cell concentration and H_2_O_2_ specific death rate (b,c) and initial hydrogen peroxide concentration and cell-specific detoxification rate (e,f). |

References

Dennis, B., E. L. Crow, K. Shimizu, and G. P. Patil. 1987. Lognormal distributions,.

Koch, A. L. 1966. The logarithm in biology 1. Mechanisms generating the log-normal distribution exactly. J Theor Biol 12: 276–290. doi:10.1016/0022-5193(66)90119-6

Limpert, E., W. Stahel, M. A.- BioScience, and undefined 2001. 2001. Log-normal distributions across the sciences: keys and clues: on the charms of statistics, and how mechanical models resembling gambling machines offer a link to a. Bioscience 51: 341–352.
